# Thermalization without Detailed Balance: Population Oscillations in the Absence of Coherences

**DOI:** 10.1021/acs.jpclett.5c00499

**Published:** 2025-04-16

**Authors:** Shay Blum, David Gelbwaser-Klimovsky

**Affiliations:** † Schulich Faculty of Chemistry and Helen Diller Quantum Center, Technion-Israel Institute of Technology, Haifa 3200003, Israel; ‡ Physics Department, Technion-Israel Institute of Technology, Haifa 3200003, Israel

## Abstract

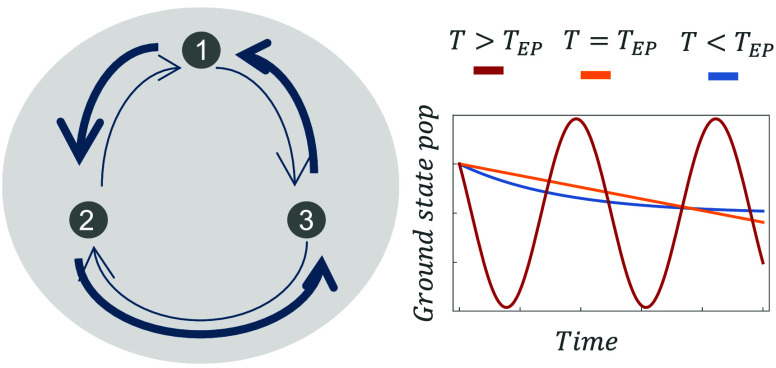

Open quantum systems that comply with the master equation and detailed balance decay in a non-oscillatory manner to thermal equilibrium. Beyond the weak coupling limit, systems that break microreversibility (e.g., in the presence of magnetic fields) violate detailed balance but still thermalize. We study the thermalization of these systems and show that a temperature increase produces novel exceptional points that indicate a sharp transition in the thermalization dynamics. A further temperature increase fuels oscillations of the energy level populations, even without quantum coherences. Moreover, the violation of detailed balance introduces an energy scale that characterizes the oscillatory regime at high temperatures.

Thermalization is the irreversible evolution toward a thermal equilibrium distribution. This process establishes a clear direction of time, even though the underlying dynamic equations are reversible in time. The emergence of irreversibility and thermalization from a reversible evolution has been the source of multiple discussions.^1−3^ For quantum systems, thermalization was first studied by Pauli.^4^ Starting from the reversible Schrödinger equation and assuming random phases, Pauli used perturbation theory to derive a rate equation for the energy level populations that explained how a quantum system in contact with its surroundings irreversibly reaches a thermal distribution. Since then, the Pauli master equation has been the standard equation for describing the thermalization of open quantum systems.^5,6^

Detailed balance (DB) has been considered as a necessary condition for thermalization,^7,8^ hampering the study of thermalization beyond DB. The role of DB in thermalization becomes evident in the Pauli rate equation. In this equation, the constraints DB imposes on the transition rates force the system to evolve toward a thermal steady state through a non-oscillatory exponential decay of the energy levels’ population. This is achieved by balancing the probability flow between each pair of eigenstates. Moreover, DB ensures the lack of persistent probability and heat currents at thermal equilibrium.^9^

Nevertheless, several systems can violate DB at equilibrium: photonic crystals,^10^ nonreciprocal planar slabs,^11^ and electrons in quantum rings,^12^ to mention a few examples. As recently has been shown in the context of Lindblad and Pauli rate equations,^13^ when these systems interact only with a thermal bath, the steady state is a thermal equilibrium state despite the violation of detailed balance at equilibrium (VDB). Systems that violate DB present interesting features at thermal equilibrium such as persistent currents,^14^ repulsive Casimir forces^15^ and potential violations of Earshaw’s theorem.^16^ However, these studies focus on systems at thermal equilibrium without investigating the thermalization process itself. In contrast, in the realm of algorithms, the VDB has been shown to accelerate the convergence of Markov chain Monte Carlo methods.^17^ This suggests the potential for VDB to influence thermalization of real physical systems, a topic that remains unexplored.

In this Letter, we use the Lindblad equation to study the thermalization of a nondegenerate *N*-level open quantum system that violates DB. We show that the VDB enables different types of dynamics that are absent for systems that keep DB, and therefore, they were unknown in the context of thermalization. The new thermalization dynamics are oscillatory decay and polynomial decay. The latter is produced by the presence of novel Liouvillian exceptional points (LEPs)^18^ in the rate equations. We show that the resulting dynamics depends on the balance between the strength of two types of processes that the system undergoes simultaneously: relaxation and a tendency to oscillate in close loops along energy state populations. As we explain below, the latter process is favored at high temperatures and a strong VDB.

LEPs have been widely studied in settings with an external driving^19,20^ or in the presence of multiple baths^18,21^ and have been shown to accelerate relaxation dynamics.^22^ Here, we find LEPs in a different scenario: an open quantum system without being driven and interacting with a single thermal bath. To the best of our knowledge, this is the first work on the effects of LEPs on thermalization in an equilibrium setup at non-zero temperatures. The LEPs in our system are a consequence of the VDB, and they set a temperature, *T*_EP_, at which there is a sharp transition from a non-oscillatory to an oscillatory decay to the thermal state.

Although energy level oscillations can be found in several systems, they are damped at high temperatures^23^ and require quantum coherences (in the eigenbasis of the system Hamiltonian).^24^ The connection between population oscillations and quantum coherences has been considered as a signature of quantumness in a wide range of fields such as open quantum systems,^25^ quantum thermodynamics,^26^ quantum biology,^27^ and quantum transport.^28^ The oscillations we study are different for two fundamental reasons. (i) They do not require quantum coherences between energy levels; (ii) they arise only when *T* > *T*_EP_ because they require the injection of energy that can only be provided by a high-temperature bath.

Finally, we study the thermalization of a toy model composed of a single electron tunneling among three quantum dots in the presence of a magnetic field.^29^ This model allows us to derive analytically the required physical conditions for decaying oscillations. Moreover, in this model, we show that the VDB introduces an energy scale, , that determines the regime of oscillatory behavior at high temperatures.

We start by considering a nondegenerate *N*-level system interacting with a single thermal bath at inverse temperature β = 1/*k*_B_*T*. The reduced dynamics follows the Gorini, Kossakowski, Lindblad, and Sudarshan (GKLS) equation.^30,31^ The latter is approximately valid in the weak coupling, low-density, or singular coupling limit.^32^ We further assume that the *N*-level system is not nearly degenerate, therefore ensuring the accuracy of the global GKLS equation instead of the local one.^33^ The coherences and population dynamic equations decoupled from each other, and the Pauli rate equation describes the population evolution:
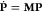
1where **P** is a vector composed of the populations of the system energy levels and **M** is the transition rate matrix with components *M*_*k*≠*l*_ = *a*_*kl*_ and *M*_*kk*_ = –*∑*_*l*≠*k*_*a*_*lk*_. *a*_*kl*_ represents the transition rate from state *l* to *k* and is derived from microscopic dynamics. For systems that are weakly coupled or maintain microreversibility, *a*_*kl*_ obeys DB,^34^ that is, , where  is the energy of the system’s level *k*. Beyond the weak coupling limit, systems that do not maintain microreversibility (e.g., systems in the presence of magnetic fields) may violate DB, but they still relax to a thermal state.^13^ Their transition rates comply with more complex constraints known as thermalization conditions or complex balancing.^35^

Despite the stationary state being the same for transition rates that keep or violate DB, how the thermal state is reached is fundamentally different. Systems that violate DB simultaneously experience two types of processes: (i) standard dissipation, which relaxes the system state toward the thermal state, reducing the relative entropy between these two states (the strength of this process is characterized by ω_dis_ = *∑*_*k*≠*l*_*a*_*kl*_ > 1/*t*_dis_, where *t*_dis_ is the thermalization time scale), and (ii) a tendency to oscillate along closed loops among the population states, for example, |*l*⟩ → |*k*⟩ → |*i*⟩ → ... → |*j*⟩ → |*l*⟩. We emphasize that these are oscillations of the system Hamiltonian eigenstates and do not require the presence of quantum coherences among these levels. These oscillations are generally absent in scenarios without driving or multiple thermal baths because they require the VDB. This is demonstrated by the violation of Kolmogorov’s criterion:^36^*c* = *a*_*lj*_ ... *a*_*ik*_*a*_*kl*_ – *a*_*lk*_*a*_*ki*_ ... *a*_*jl*_ ≠ 0, indicating a difference between the forward and backward processes, and implying a non-zero affinity.^37^ On multilevel systems, there could be several different closed loops among population states. DB implies *c* = 0 for all possible closed loops and, therefore, no oscillations. As we show below, the balance between the strengths of the two types of processes (|*c*| vs ω_dis_) determines the thermalization dynamics.

The general solution of eq 1 is
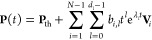
2where **P**_th_ is the thermal distribution and *b*_*i*,*l*_ are parameters set by the initial conditions. λ_*i*_ and **V**_*i*_ are the eigenvalues and generalized eigenvectors of **M**, respectively. *d*_*i*_ is the number of generalized eigenvectors related to eigenvalue λ_*i*_ (see section I of the Supporting Information). If the transition rates maintain DB, then all λ_*i*_ values are real and nonpositive, and *d*_*i*_ = 1. The system is thermalized through a linear combination of decaying exponentials. We term this dynamics non-oscillatory decay. If the transition rates violate DB, the thermalization dynamics can be different. Besides the non-oscillatory decay, there are two other dynamical regimes. (1) For large enough values of *c* and temperature, some of the eigenvalues become complex with a nonpositive real part, and the dynamics acquires an oscillatory decay component. Here too *d*_*i*_ = 1. Although these oscillations may be used for a transient work extraction, it is based on the resources present in the initial state. The lack of steady work extraction ensures compliance with the second law of thermodynamics. (2) The regime between oscillatory and non-oscillatory decay is divided by the exceptional point dynamics. In this case, **M** has some real nonpositive eigenvalues that are degenerate, and their respective eigenvectors coalesce. **M** can no longer be diagonalized, and its Jordan form is required for the derivation of eq 2 (*d*_*i*_ > 1 for at least one *i*). The relaxation to the thermal state acquires a polynomial component that multiplies the exponential decay. Exceptional points of **M** are termed LEPs^18^ because they are related to the Liouvillian quantum dynamics of open systems, rather than the Hamiltonian dynamics. Reaching all of the dynamical regimes is not ensured by the VDB. Physical transition rates are limited by the thermalization conditions. As shown below, even under those constraints, quantum systems can experience an LEP and oscillatory thermalization dynamics.

The temperature and strength of the VDB determine the thermalization dynamics. Because the system is nondegenerate, oscillations in close loops along the populations need energy that can only be provided by the bath. If the temperature is low, the bath cannot supply the required energy and the system relaxes exponentially to equilibrium with no oscillations (see section VI of the Supporting Information). For higher temperatures, the bath can fuel the oscillations. In this case, if the VDB is strong enough (i.e., large |*c*|), then the thermalization dynamics will exhibit an oscillatory decay. To determine the required amount of the VDB for triggering oscillations, we study the simplest open system that can violate DB without driving or multiple baths: an open three-level system toy model.

For this, we consider three quantum dots (3QDs) in an equilateral triangle arrangement under the influence of a magnetic field^29^ (see Figure 1). **r**_*i*_ are the QDs positions. Assuming there is only a single electron in the system, its Hamiltonian in the single-electron localized basis (|1⟩, |2⟩, |3⟩) is
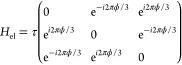
3where τ is the tunneling constant and ϕ is the magnetic flux quanta, which allows the breaking of microreversibility. The eigenenergies of this Hamiltonian are . The 3QDs interact with a low-density gas of free particles with mass *m*. The gas is in a thermal state with inverse temperature β = 1/*k*_B_*T*. We assume that if there is an electron in the quantum dot, a nearby particle will feel a short-range repulsive potential. In particular, we model the interaction Hamiltonian as *H*_int_ = ∑_*i*∈{1,2,3}_*V*_*i*_δ(**r** – **r**_*i*_)|*i*⟩⟨*i*|. Microreversibility and DB are recovered if *V*_*i*_ = *V*_*j*_ for any pair with *i* ≠ *j*. The low density of the gas allows one to describe the reduced dynamics of the 3QDs with the low-density limit GKLS equation. At this limit, the gas statistic does not play any role.^38^ Here, the role of the jump operators is fulfilled by the on shell -matrix elements describing the scattering of the low-density gas particles by the electron of the 3QDs.  represents the probability for a process that starts with the 3QDs in state *l* and a gas particle with momenta **p** and ends with the 3QDs in state *k* and the scattered gas particle with momenta **p′**. For a short separation between the three quantum dots and a 1D gas, the -matrix elements can be calculated analytically, and from them, the transition rates are obtained by tracing out the incoming and outgoing gas particle momenta (see section IV of the Supporting Information):
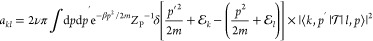
4where *Z*_P_ is the partition function of the gas particle and ν is the gas density. These transition rates derived from a physical microscopic model are used to build the transition rate matrix **M**.

**Figure 1:**
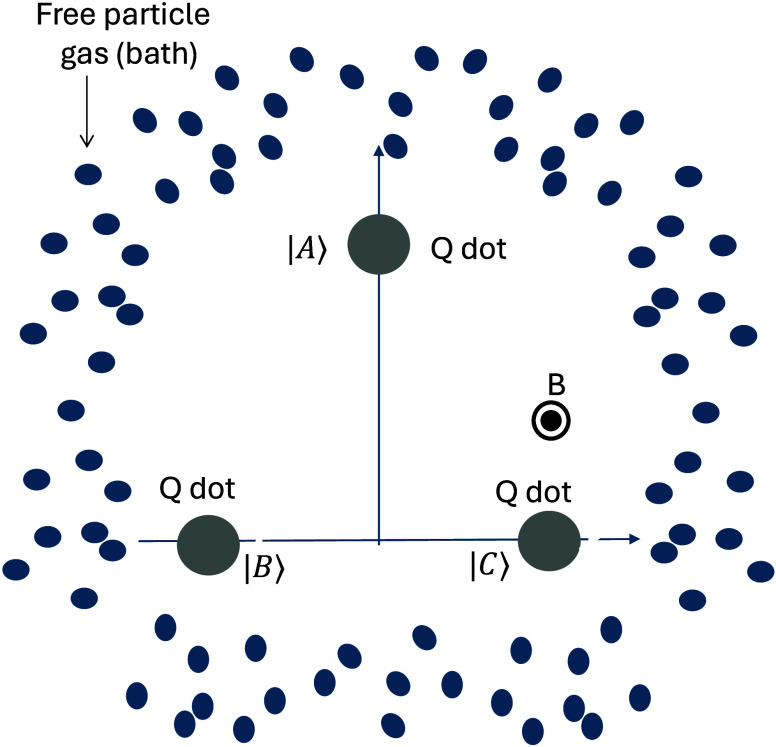
Toy model. A free particle gas (thermal bath) is scattered by a single electron that tunnels among three QDs in a magnetic field. In this work, we assume a short separation between the three quantum dots and a 1D gas. These assumptions allow us to calculate analytically the -matrix elements.

Figure 2 shows the number of oscillations during the system thermalization time scale as a function of temperature *T* and *V*_1_. *V*_1_ determines the strength of the VDB and the asymmetry of the dynamics, which determine the oscillation direction. *T* fixes the bath energy that drives the oscillations. The number of oscillations indicates the different dynamical regimes and is determined by the ratio between the imaginary and real parts of the **M** non-zero eigenvalue. As discussed above, the system always decays without oscillations to the thermal state at low temperatures. This changes at higher temperatures. If the VDB is large enough, there is a temperature, *T*_EP_, at which a LEP is formed and a sharp transition on the thermalization dynamics takes place. Above *T*_EP_, the system thermalizes through decaying oscillations. Notice that the regions that comply with DB (i.e., *V*_1_ = *V*_2_ or *V*_1_ = *V*_3_) divide between oscillatory decay with different directions: clockwise in the middle region (|−⟩ → |0⟩ → |+⟩ → |−⟩) where the rates in the clockwise direction (*a*_0–_, *a*_+0_, and *a*_–+_) are the largest rates and counterclockwise in the lateral regions (|−⟩ → |+⟩ → |0⟩ → |−⟩) where the counterclockwise direction rates are the dominant rates (*a*_+–_, *a*_0+_, and *a*_–0_). The plotted oscillation number never exceeds 0.16. However, the oscillation number can be increased by considering larger systems^39^ and increasing the strength of the VDB. It can reach one for a four-level system and surpass two for a seven-level system (see section VII of the Supporting Information).

**Figure 2:**
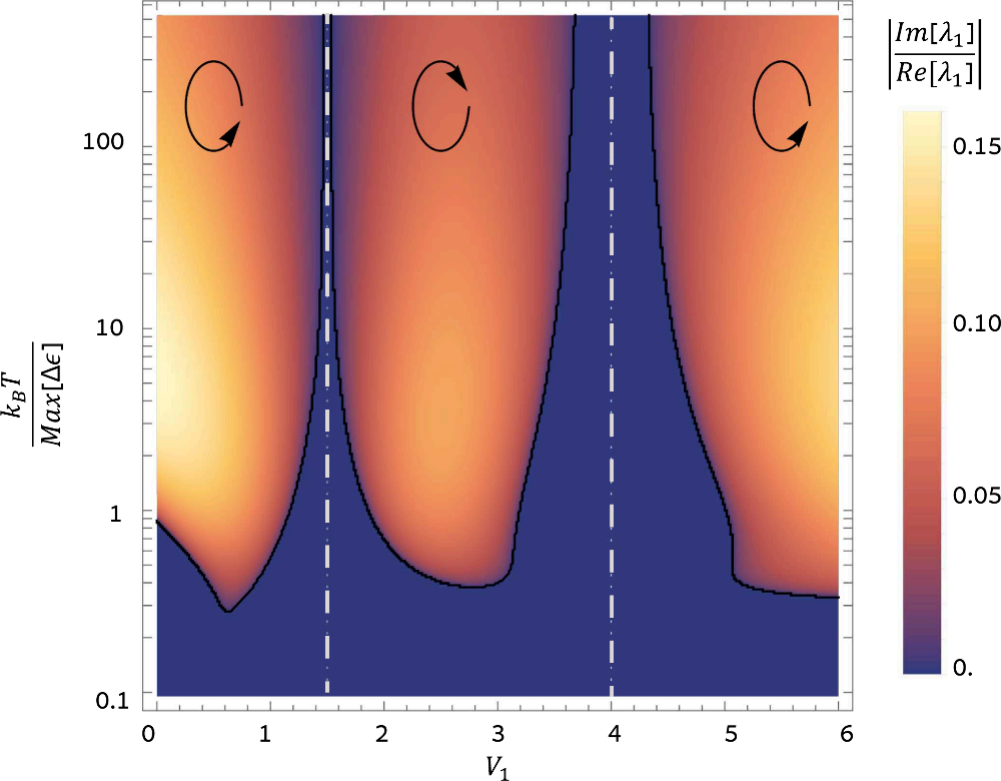
3QD thermalization regimes as a function of *V*_1_ (*x*-axis) and temperature *T* (*y*-axis). *V*_1_ controls the VDB. The plot shows the absolute value of the ratio between the imaginary and real part of the non-zero eigenvalue of **M**. For non-oscillatory decay (blue regions), eigenvalues are real and the plotted rate is zero. For oscillatory decay (yellow/orange regions), eigenvalues can be complex and the plotted rate gets a non-zero value. Black lines separating oscillatory and non-oscillatory decay correspond to the exceptional point dynamics and determine *T*_EP_. Gray dashed vertical lines correspond to regions where microreversibility and DB are reestablished (*V*_1_ = *V*_2_ = 1.5, and *V*_1_ = *V*_3_ = 4). Parameters: τ = 1.85, ϕ = 0.575, and *ℏ* = *k*_B_ = *m* = 1. Max .

The VDB or oscillation strength required to trigger the oscillatory decay can be determined by analyzing the eigenvalues of **M**. The oscillation strength is the difference between the rate of the counterclockwise and clockwise processes, i.e., *c* = *a*_–+_*a*_+0_*a*_0–_ – *a*_–0_*a*_0+_*a*_+–_. To have oscillatory decay, *c* should maintain the inequality (see section II of the Supporting Information)
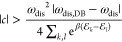
5where  is the sum of the respective rates when DB holds. Equation 5 confirms the physical intuition that the balance between oscillations and dissipation strengths, |*c*| and ω_dis_, respectively, determines the thermalization dynamics. Equation 5 becomes an equality at the LEP.

The mechanism driving the system to an oscillatory decay can be understood by analyzing the rates and the corresponding  matrix. For our system, the rates (eq 4) can be rewritten in the following form, which simplifies the analysis (see section IV of the Supporting Information):
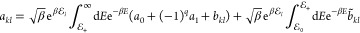
6where *q* = 0 for clockwise rates and *q* = 1 otherwise. The terms *a*_0_, *a*_1_, *b*_*kl*_, and  are independent of the temperature. Oscillatory decay takes place at high temperatures. Increasing the temperature increases the number of gas particles with high energy, which eventually can provide the dominant contribution to *a*_*kl*_. Therefore, the mechanism behind the oscillatory dynamics can be understood from the high-energy expansion of *a*_0_, *a*_1_, and *b*_*kl*_ (see section IV of the Supporting Information):
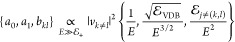
7where  is the energy scale related to the VDB. In terms of the Hamiltonian parameters, , where *v*_*kl*_ = ∑_*i*=1_^3^⟨*k*|*i*⟩*V*_*i*_⟨*i*|*l*⟩, *k*, *l* ∈ {+, 0, −} are the matrix elements of the interaction Hamiltonian part that acts on the system. They have units of energy × length. For our system, |*v*_*kl*_|^2^ is the same for any *k* ≠ *l*, and therefore  is well-defined. Equation 7 shows that at high energies the main contribution to the rates is *a*_0_. *a*_1_ and *b*_*kl*_ are first- and second-order corrections, respectively. For , *a*_0_ does not distinguish between transitions unless the interaction Hamiltonian has some asymmetry such that |*v*_*kl*_|^2^ ≠ |*v*_*lm*_|^2^ (*k* ≠ *l* ≠ *m*). This makes *a*_0_ transition independent for our model. The high energy term of *a*_0_ originates from the Born or weak coupling approximation of the -matrix, which does not contribute to the VDB.^13^ The VDB only arises at the next order of the -matrix Born series that is proportional to
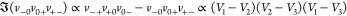
8(see section III of the Supporting Information). This quantity is the Hamiltonian equivalent to the difference between the clockwise and counterclockwise processes and is proportional to the violation of microreversibility. This -matrix term produces the high energy limit of (−1)^*q*^*a*_1_, which does not distinguish among all of the individual transitions but makes a difference between clockwise and counterclockwise rates through the prefactor (−1)^*q*^. Next, there is *b*_*kl*_, which is the first term in the 1/*E* series expansion that distinguishes between transitions in the same direction, but not between directions, i.e., *b*_*kl*_ = *b*_*lk*_. Finally, the  integral is limited to , so it does not include high-energy contributions.

If the VDB is large enough (see eq 5) at high temperatures the high-energy contributions of *a*_1_ will overshadow the low-energy contributions of *b*_*kl*_ and . In this case, the rates in the same direction have approximately the same value:
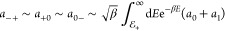
9 and
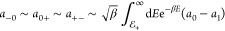
10Under these circumstances, thermalization occurs through decaying oscillations with a frequency proportional to  (see Figure 3). For weak VDB, the rates at high temperatures do not group into two different values depending on their direction and thermalization takes place through non-oscillatory relaxation (regions around *V*_1_ ∼ *V*_2_ = 1.5 and *V*_1_ ∼ *V*_3_ = 4 in Figure 2).

**Figure 3:**
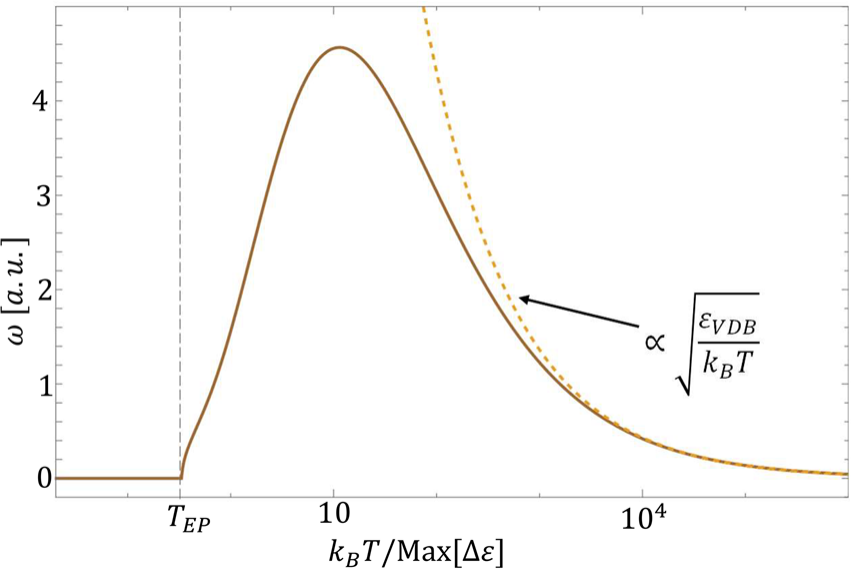
Population oscillation frequency during thermalization (solid brown line) as a function of the bath temperature for a strong VDB (see eq 5). At low temperatures, the system thermalizes through a non-oscillatory decay. When *T* = *T*_EP_, there is a LEP, which produces a sharp transition to a thermalization through decaying oscillations. At high temperatures, the oscillation frequency is proportional to  (dashed yellow line), where  is the VDB energy scale (see eq 7). Parameters: the same as Figure 2 with *V*_1_ = 6.

Finally, we derive a sufficient but not necessary condition for oscillatory decay at the high-temperature limit, i.e., . For this, we introduce an energy scale , which is related to low-energy processes (i.e., , which are related to  and processes of order ; see eq 7). At high temperatures, if
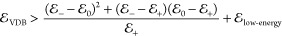
11the thermalization dynamics has to be through decaying oscillations. This inequality was derived using the high-energy expansion of the rate processes at the high-temperature limit (see section V of the Supporting Information). For lower temperatures, Boltzmann factors in the rates cannot be neglected, complicating the derivation of a compact energetic condition as eq 11.

In summary, the VDB produces alternative thermalization paths that result in two phenomena: (i) the existence of LEPs under equilibrium conditions (i.e., without driving and in the presence of a single thermal bath). These novel LEPs produce a sharp transition in the thermalization dynamics, triggering oscillations. These LEPs could be used to expand the EPs’ advantages for sensing^40,41^ to thermal equilibrium settings, allowing the creation of more precise measurement protocols of equilibrium variables such as temperature. This will require overcoming the impact of noise.^42,43^ (ii) Unique energy level population oscillations without quantum coherence that instead of being damped they are fueled by high-temperature thermal noise. At high temperatures, the frequency of these oscillations, ω, is determined by the VDB natural energy scale, : . Moreover, the relative value of  to other energy scales sets conditions for oscillations.

## Supporting Information Available

Additional details about (1) the general solution to the Pauli master equation, (2) Eigen decomposition of the transition rate matrix and condition for oscillations, (3) derivation of rates of transitions and the -matrix elements, (4) decomposition of the rates of transition, (5) sufficient condition for oscillations at the high-temperature limit, (6) proof for no oscillations in the low-temperature limit, and (7) the *N*-level system toy model (PDF)
Transparent Peer Review report available (PDF)
