# Supplementary material for: Thermalization without Detailed Balance: Population Oscillations in the Absence of Coherences
Source: J Phys Chem Lett. 2025 Apr 16;16(16):4066–71. doi: 10.1021/acs.jpclett.5c00499 (PMC12035852; doi:10.1021/acs.jpclett.5c00499)
Supplement: Supplementary file 1 — jz5c00499_si_001.pdf [file jz5c00499_si_001.pdf]

SI - Thermalization without detailed balance: population oscillations in the absence of coherences

Shay Blum[1,2] and David Gelbwaser-Klimovsky[1,*]

[1]Schulich Faculty of Chemistry and Helen Diller Quantum Center, Technion-Israel Institute of Technology, Haifa 3200003, Israel

[2]Physics Department, Technion-Israel Institute of Technology, Haifa, 3200003, Israel

I. GENERAL SOLUTION TO THE PAULI MASTER EQUATION

The solution for equation (1) in the main text is given by: $\mathbf{P}(t) = e^{Mt}\mathbf{P}_0$ where $\mathbf{P}_0 = \mathbf{P}(t=0)$ sets the initial conditions. In order to arrive at equation (2) in the main text, one should consider the Jordan canonical form J of the matrix M. Generally it has the form $J = diag\{J_1, J_2, ..., J_\kappa\}$ where J_i are Jordan blocks related to eigenvalue λ_i, with dimension d_i. Note that by definition $\sum_{i=1}^{\kappa} d_i = N$, where N is the dimension of M.
Considering the basis transfer matrix Q, we have:

$$\mathbf{P}(t) = e^{Mt}\mathbf{P}_0 = e^{QJQ^{-1}t}\mathbf{P}_0 = Qe^{Jt}Q^{-1}\mathbf{P}_0 \tag{S.I.1}$$

From the structure of J, we have $e^{Jt} = diag\left\{e^{J_1 t}, e^{J_2 t}, ..., e^{J_\kappa t}\right\}$, where the exponential of a Jordan block is

$$\left(e^{J_i t}\right)_{k\ell} = e^{\lambda_i t}\begin{cases}\frac{1}{(\ell-k)!}t^{\ell-k} & k \leq \ell \\ 0 & k > \ell\end{cases} \tag{S.I.2}$$

Denoting $\tilde{\mathbf{P}}(t) = Q^{-1}\mathbf{P}(t)$, we can define the vectors $\tilde{\mathbf{P}}_0^n$ by

$$\left(\tilde{\mathbf{P}}_0^n\right)_i = \begin{cases}\left(\tilde{\mathbf{P}}_0\right)_i & \sum_{s=1}^{n-1} d_s < i \leq \sum_{s=1}^{n} d_s \\ 0 & \text{else}\end{cases} \tag{S.I.3}$$

such that $\tilde{\mathbf{P}}_0 = \sum_{n=1}^{\kappa}\tilde{\mathbf{P}}_0^n$ and

$$\tilde{\mathbf{P}}(t) = e^{Jt}\tilde{\mathbf{P}}_0 = \sum_{n=1}^{\kappa} e^{Jt}\tilde{\mathbf{P}}_0^n = \sum_{n=1}^{\kappa} e^{\bar{J}_n t}\tilde{\mathbf{P}}_0^n \tag{S.I.4}$$

where $\bar{J}_n = diag\{0, 0, ..., 0, J_n, 0..., 0\}$ sets all blocks in J to be zero except for J_n. For calculating $e^{\bar{J}_n t}\tilde{\mathbf{P}}_0^n$ we consider the following:
Let J_0 be a Jordan block related to an eigenvalue λ_0 with dimension d_0, and let $\mathbf{\Pi}$ be some vector of dimension d_0. Then:

$$e^{J_0 t}\mathbf{\Pi} = \sum_{i=1}^{d_0}\hat{e}_i\left(\sum_{j=1}^{d_0}\left(e^{J_0 t}\right)_{ij}\Pi_j\right) = e^{\lambda_0 t}\sum_{i=1}^{d_0}\hat{e}_i\left(\sum_{j=i}^{d_0}\frac{1}{(j-i)!}t^{j-i}\Pi_j\right) \tag{S.I.5}$$

with $(\hat{e}_i)_j = \delta_{ij}$ being the standard basis vectors. From this we conclude:

$$\tilde{\mathbf{P}}(t) = \sum_{n=1}^{\kappa} e^{\lambda_n t}\sum_{i=1}^{d_n}\hat{e}_i^n\left(\sum_{j=i}^{d_n}\frac{1}{(j-i)!}t^{j-i}\left(\tilde{\mathbf{P}}_0^n\right)_j\right) \tag{S.I.6}$$

---

* dgelbi@technion.ac.il

with $\hat{e}_i^n = \hat{e}_{i+\sum_{s=1}^{n-1} d_s}$, a standard basis vector shifted to block n.
In terms of $\mathbf{P}(t)$ we use the generalized eigenvectors $\mathbf{V}_i^n = Q\hat{e}_i^n$ for block n and have the complete expression:

$$\mathbf{P}(t) = \sum_{n=1}^{\kappa}\sum_{i=1}^{d_n} e^{\lambda_n t}\mathbf{V}_i^n \left(\sum_{j=i}^{d_n} \frac{1}{(j-i)!} t^{j-i} \left(\tilde{\mathbf{P}}_0^n\right)_j\right) \tag{S.I.7}$$

For M that is given in equation (1) in the main text, there is a single eigenvalue that is $\lambda_1 = 0$, i.e., $d_1 = 1$, with corresponding eigenvector $\mathbf{V}_1^1 = \mathbf{P}_{th}$. Hence:

$$\mathbf{P}(t) = \mathbf{P}_{th} + \sum_{n=2}^{\kappa}\sum_{i=1}^{d_n} e^{\lambda_n t}\mathbf{V}_i^n \left(\sum_{j=i}^{d_n} \frac{1}{(j-i)!} t^{j-i} \left(\tilde{\mathbf{P}}_0^n\right)_j\right) \tag{S.I.8}$$

In (S.I.8), we sum over $N-1$ generalized eigenvectors $\mathbf{V}_i^n$ (excluding $\mathbf{P}_{th}$), where each one is multiplied by polynomial in t, with maximum degree of $d_n - 1$. If we rename the indices by going over all generalized eigenvectors and changing $\{\mathbf{V}_i^n, \lambda_n, d_n\} \to \{\mathbf{V}_s, \lambda_s, d_s\}$ s.t. we allow repetitions in λ_s and d_s is the dimension of the block related to $\mathbf{V}_s$, then the expression can be written as:

$$\mathbf{P}(t) = \mathbf{P}_{th} + \sum_{s=2}^{N}\sum_{\ell=0}^{d_n-1} b_{s\ell} t^{\ell} e^{\lambda_s t}\mathbf{V}_s \tag{S.I.9}$$

where $b_{s\ell}$ can be zero and depend on $\tilde{\mathbf{P}}_0$. Therefore, $b_{s\ell}$ are related to the initial conditions.

II. EIGEN-DECOMPOSITION OF THE TRANSITION RATE MATRIX AND CONDITION FOR OSCILLATIONS

The eigenvalues of a transition rate matrix for a 3-level system are:

$$\begin{aligned} \lambda_0 &= 0 \\ \lambda_\pm &= -\frac{1}{2}\omega_{dis} \pm \frac{1}{2}\sqrt{\gamma} \end{aligned} \tag{S.II.1}$$

where

$$\begin{aligned} \omega_{dis} &= \sum_{i\neq j} a_{ij} \\ \gamma &= \omega_{dis}^2 - 4\left(a_{-0}a_{-+} + a_{-+}a_{0-} + a_{-0}a_{0+} + a_{0-}a_{0+} + \right. \\ &\quad \left. + a_{-0}a_{+-} + a_{0+}a_{+-} + a_{-+}a_{+0} + a_{0-}a_{+0} + a_{+-}a_{+0}\right) \end{aligned} \tag{S.II.2}$$

The corresponding eigenvectors are:

$$\begin{aligned} \mathbf{V}^0 &= \begin{pmatrix} a_{-0}a_{-+} + a_{-0}a_{0+} + a_{-+}a_{+0} \\ a_{0+}a_{0-} + a_{0+}a_{+-} + a_{0-}a_{-+} \\ a_{+-}a_{+0} + a_{+-}a_{-0} + a_{+0}a_{0-} \end{pmatrix} \\ \mathbf{V}^\pm &= \begin{pmatrix} \alpha_1 + \alpha_2 \pm \sqrt{\gamma} \\ \alpha_1 - \alpha_2 \mp \sqrt{\gamma} \\ -2\alpha_1 \end{pmatrix} \end{aligned} \tag{S.II.3}$$

where

$$\begin{aligned} \alpha_1 &= a_{+0} - a_{+-} \\ \alpha_2 &= -a_{-0} + a_{-+} - a_{0-} + a_{0+} \end{aligned} \tag{S.II.4}$$

As we can see, we have a single zero eigenvalue, λ_0. Its respective eigenvector is proportional to the steady state vector $\mathbf{V}^0 \propto \mathbf{P}_{th}$.
Assuming a thermal stationary state, then $\mathbf{P}_{th}$ describes the Boltzmann distribution:

$$\mathbf{P}_{th} = \frac{1}{Z_S}\begin{pmatrix} e^{-\beta\mathcal{E}_-} \\ e^{-\beta\mathcal{E}_0} \\ e^{-\beta\mathcal{E}_+} \end{pmatrix} \tag{S.II.5}$$

With this assumption, requiring $M\mathbf{P}_{th}=0$ one gets the thermalization conditions:

$$
\begin{aligned}
a_{0-}\left(I_{-0}-1\right)+a_{+-}\left(I_{-+}-1\right)&=0\\
a_{-0}\left(I_{0-}-1\right)+a_{+0}\left(I_{0+}-1\right)&=0\\
a_{-+}\left(I_{+-}-1\right)+a_{0+}\left(I_{+0}-1\right)&=0
\end{aligned}
\tag{S.II.6}
$$

where we define $a_{ij}e^{-\beta\mathcal{E}_j}=a_{ji}e^{-\beta\mathcal{E}_i}I_{ij}$. Noting that $I_{ij}=I_{ji}^{-1}$, we can write:

$$
\begin{aligned}
I_{-0}&=\frac{a_{+0}\left(I_{0+}-1\right)e^{\beta(\mathcal{E}_{-}-\mathcal{E}_0)}}{a_{0-}}+1\\
I_{+-}&=\frac{a_{+0}\left(I_{0+}-1\right)e^{\beta(\mathcal{E}_{+}-\mathcal{E}_0)}}{a_{-+}}+1
\end{aligned}
\tag{S.II.7}
$$

Plugging these relations on γ we get:

$$
\gamma=\omega_{dis}^2-4\frac{c}{\omega_{dis,DB}-\omega_{dis}}\sum_{i,j}e^{\beta(\mathcal{E}_i-\mathcal{E}_j)}
\tag{S.II.8}
$$

where

$$
\begin{aligned}
\omega_{dis}&=a_{-+}\left(e^{\beta(\mathcal{E}_{-}-\mathcal{E}_{+})}+1\right)+a_{0-}\left(e^{\beta(\mathcal{E}_0-\mathcal{E}_{-})}+1\right)+a_{+0}\left(I_{0+}\left(1+e^{\beta(\mathcal{E}_{-}-\mathcal{E}_0)}+e^{\beta(\mathcal{E}_{+}-\mathcal{E}_0)}\right)-e^{\beta(\mathcal{E}_{-}-\mathcal{E}_0)}\right)\\
\omega_{dis,DB}&=a_{-+}\left(e^{\beta(\mathcal{E}_{-}-\mathcal{E}_{+})}+1\right)+a_{0-}\left(e^{\beta(\mathcal{E}_0-\mathcal{E}_{-})}+1\right)+a_{+0}\left(1+e^{\beta(\mathcal{E}_{+}-\mathcal{E}_0)}\right)
\end{aligned}
\tag{S.II.9}
$$

and we use that $\operatorname{sign}(c)=\operatorname{sign}(\omega_{dis,DB}-\omega_{dis})$.
The condition for oscillations is demanding $\gamma<0$, thus becoming equivalent to the one presented in equation (5) in the main text.

$$
|c|>\frac{\omega_{dis}^2\left|\omega_{dis,DB}-\omega_{dis}\right|}{4\sum_{i,j}e^{\beta(\mathcal{E}_i-\mathcal{E}_j)}}=\frac{\omega_{dis}^2\left|\omega_{dis,DB}-\omega_{dis}\right|}{4\left(3+\sum_{i\neq j}e^{\beta(\mathcal{E}_i-\mathcal{E}_j)}\right)}
\tag{S.II.10}
$$

Note that for oscillations with $\gamma<0$, the oscillations number during the system thermalization time scale is defined as the ratio between the imaginary and real part of the non-zero eigenvalues, i.e., $|\sqrt{\gamma}/\omega_{dis}|$. We can bound the square of this quantity by taking only the negative terms in γ:

$$
\frac{|\gamma|}{\omega_{dis}^2}<2\frac{a_{-0}\left(a_{-+}+a_{0+}+a_{+-}\right)+a_{-+}\left(a_{0-}+a_{+0}\right)+\left(a_{0-}+a_{+-}\right)\left(a_{0+}+a_{+0}\right)}{\left(\sum_{i\neq j}a_{ij}\right)^2}<1
\tag{S.II.11}
$$

III. DERIVATION OF RATES OF TRANSITIONS AND THE T MATRIX ELEMENTS

A. The Low-Density Limit in Open Quantum Systems

In the low-density limit the quantum master equation is derived for a discrete level system coupled to a thermal bath of free particles. The local interaction between the gas and the system describes scattering processes where the gas density is taken to be low enough such that we can consider each scattering process to be independent from one another. Therefore, these processes involve only a single particle scattering process [1].
The effective Hamiltonian is:

$$
H=H_P\otimes 1+1\otimes H_S+H_{int}
\tag{S.III.1}
$$

where

$$
\begin{aligned}
H_P&=\int d\boldsymbol{p}E_{\boldsymbol{p}}\left|\boldsymbol{p}\right\rangle\left\langle\boldsymbol{p}\right|\\
H_S&=\sum_j\mathcal{E}_j\left|j\right\rangle\left\langle j\right|
\end{aligned}
\tag{S.III.2}
$$

H_P being the free particle Hamiltonian, with $E_{\boldsymbol{p}} = \frac{\boldsymbol{p}^2}{2m}$, and H_S being the system Hamiltonian. We denote also $H_0 = H_P \otimes 1 + 1 \otimes H_S$ for later use.
When deriving the quantum master equation, the particle's momentum distribution is given by Boltzmann distribution:

$$G(\boldsymbol{p}) = \frac{e^{-\beta E_{\boldsymbol{p}}}}{Z_P} \tag{S.III.3}$$

The master equation then takes the form:

$$\frac{d}{dt}\rho_S = (\mathcal{L}_S + \mathcal{L}_D)\,\rho_S \tag{S.III.4}$$

where $\mathcal{L}_S = -i\,[H_S, \cdot]$ is the system's Liouvillian, describing the system's unitary evolution, and $\mathcal{L}_D$ is the dissipator:

$$\begin{aligned}\mathcal{L}_D\rho_S = \nu\pi \sum_{\omega\in \mathrm{Sp}(i\mathcal{L}_S)} \int d\boldsymbol{p}d\boldsymbol{p}' G(\boldsymbol{p})\,\delta\left(E_{\boldsymbol{p}'} - E_{\boldsymbol{p}} + \omega\right)\big(\left[T_\omega(\boldsymbol{p}',\boldsymbol{p})\,\rho_S, T_\omega^\dagger(\boldsymbol{p}',\boldsymbol{p})\right] \\ + \left[T_\omega(\boldsymbol{p}',\boldsymbol{p})\,,\rho_S T_\omega^\dagger(\boldsymbol{p}',\boldsymbol{p})\right]\big)\end{aligned} \tag{S.III.5}$$

where $\mathrm{Sp}\,(i\mathcal{L}_S)$ is the spectrum of $i\mathcal{L}_S$ (all possible eigenenergies differences). The jump operators $T_\omega(\boldsymbol{p}',\boldsymbol{p})$ are given by:

$$T_\omega(\boldsymbol{p}',\boldsymbol{p}) = \sum_{\mathcal{E}_k - \mathcal{E}_\ell = \omega} \langle \boldsymbol{p}', k\,|T|\,\boldsymbol{p},\ell\rangle\,|k\rangle\,\langle \ell| \tag{S.III.6}$$

with $\langle \boldsymbol{p}', k\,|T|\,\boldsymbol{p},\ell\rangle$ being the T-matrix element related to the scattering process $|\boldsymbol{p},\ell\rangle \to |\boldsymbol{p}',k\rangle$. For a non-degenerate H_S, the populations $P_i = \langle i\,|\rho_S|\,i\rangle$ are governed by the Pauli rate equation with transition rates given by equation (4) in the main text.

B. T matrix elements

The T-matrix can be defined as [3]:

$$T(E) = H_{int} + H_{int}G(E)\,H_{int} \tag{S.III.7}$$

with $G(E) = (E - H)^{-1}$ being the Green operator of the entire Hamiltonian. Equivalently, we can write the Lippmann-Schwinger equation for T:

$$T(E) = H_{int} + H_{int}G_0(E)\,T(E) \tag{S.III.8}$$

with $G_0(E) = (E - H_0)^{-1}$ being the Green operator of the free Hamiltonian. By introducing the Moller operators:

$$\Omega_\pm = \lim_{t\to\mp\infty} U^\dagger(t)\,U_0(t) = \lim_{t\to\mp\infty} e^{iHt}e^{-iH_0 t} \tag{S.III.9}$$

we map the asymptotic states to the state of the system at time 0:

$$\begin{aligned}\Omega_+\,|\psi_{in}\rangle = \Omega_+\,|\boldsymbol{p},\ell\rangle = |\psi\rangle \\ \Omega_-\,|\psi_{out}\rangle = \Omega_-\,|\boldsymbol{p}',k\rangle = |\psi\rangle\end{aligned} \tag{S.III.10}$$

This allows us to write the T matrix elements as:

$$\langle \boldsymbol{p}', k\,|T|\,\boldsymbol{p},\ell\rangle = \langle \boldsymbol{p}', k\,|H_{int}|\,\boldsymbol{p},\ell\rangle + \langle \boldsymbol{p}', k\,|H_{int}G_0H_{int}|\,\psi\rangle \tag{S.III.11}$$

with the energy being $E = E_{\mathbf{p}} + \mathcal{E}_\ell$.
The state $|\psi\rangle$ can be written as

$$|\psi\rangle = \Omega_+\,|\boldsymbol{p},\ell\rangle = |\boldsymbol{p},\ell\rangle + G_0 H_{int}\,|\psi\rangle \tag{S.III.12}$$

such that

$$\langle \boldsymbol{p}', k\,|T|\,\boldsymbol{p},\ell\rangle = \langle \boldsymbol{p}', k\,|H_{int}|\,\psi\rangle \tag{S.III.13}$$

C. Toy model T matrix elements

The interaction term is given by:

$$H_{int} = \sum_i V_i(\hat{\boldsymbol{q}}) |\chi_i\rangle \langle\chi_i| \tag{S.III.14}$$

where $V_i(\mathbf{q})$ are the scattering potential in each site i, and $|\chi_i\rangle$ is in the position basis of the system. The T matrix elements take the form:

$$\begin{aligned}\langle \boldsymbol{p}'j' |T| \boldsymbol{p}j\rangle &= \langle \boldsymbol{p}'j' |H_{int}| \psi\rangle \\ &= \left\langle \boldsymbol{p}'j' \middle| \left(\sum_i V_i(\hat{\boldsymbol{q}}) |\chi_i\rangle\langle\chi_i|\right) \int_{\mathbb{R}^d} \mathrm{d}^d q' \sum_k \psi_k(\boldsymbol{q}') \middle| \boldsymbol{q}'k \right\rangle = \\ &= \sum_k \sum_i \left(\langle j' \mid \chi_i\rangle \langle \chi_i \mid k\rangle\right) \int_{\mathbb{R}^d} \mathrm{d}^d q' \psi_k(\boldsymbol{q}') \langle \boldsymbol{p}' |V_i(\hat{\boldsymbol{q}})| \boldsymbol{q}'\rangle = \\ &= \sum_k \sum_i \left(\langle j' \mid \chi_i\rangle \langle \chi_i \mid k\rangle\right) \int_{\mathbb{R}^d} \mathrm{d}^d q' \psi_k(\boldsymbol{q}') \frac{e^{-\frac{i}{\hbar}\boldsymbol{p}'\cdot\boldsymbol{q}'}}{(2\pi\hbar)^{d/2}} V_i(\boldsymbol{q}')\end{aligned} \tag{S.III.15}$$

where we used:

$$\psi_j(\boldsymbol{q}) = \langle \boldsymbol{q}j \mid \psi\rangle, \quad \tilde{\psi}_j(\boldsymbol{p}) = \langle \boldsymbol{p}j \mid \psi\rangle \tag{S.III.16}$$

and:

$$\langle \boldsymbol{q}j \mid \boldsymbol{p}j'\rangle = \delta_{jj'} \frac{e^{\frac{i}{\hbar}\boldsymbol{p}\cdot\boldsymbol{q}}}{(2\pi\hbar)^{d/2}} \tag{S.III.17}$$

The wavefunction in momentum space is given by:

$$\begin{aligned}\tilde{\psi}_{j'}(\boldsymbol{p}') &= \langle \boldsymbol{p}'j' \mid \boldsymbol{p}j\rangle + \langle \boldsymbol{p}'j' |G_0 H_{int}| \psi\rangle = \\ &= \delta^d(\boldsymbol{p}-\boldsymbol{p}')\,\delta_{jj'} + \frac{\langle \boldsymbol{p}'j' |H_{int}| \psi\rangle}{E - E_{\boldsymbol{p}'} - \mathcal{E}_{j'} + i\varepsilon}\end{aligned} \tag{S.III.18}$$

where we use the geometric series expansion:

$$\langle \boldsymbol{p}'j'| G_0(E+i\varepsilon) = \langle \boldsymbol{p}'j'| \frac{1}{E - H_0 + i\varepsilon} = \langle \boldsymbol{p}'j'| \frac{1}{E - E_{\boldsymbol{p}'} - \mathcal{E}_{j'} + i\varepsilon} \tag{S.III.19}$$

This allows us to write a closed equation for the wavefunction:

$$\begin{aligned}\tilde{\psi}_{j'}(\boldsymbol{p}') = {}& \delta^d(\boldsymbol{p}-\boldsymbol{p}')\,\delta_{jj'} + \\ &+ \frac{1}{E - E_{\boldsymbol{p}'} - \mathcal{E}_{j'} + i\varepsilon} \sum_k \sum_i \left(\langle j' \mid \chi_i\rangle \langle \chi_i \mid k\rangle\right) \int_{\mathbb{R}^d} \mathrm{d}^d q' \psi_k(\boldsymbol{q}') \frac{e^{-\frac{i}{\hbar}\boldsymbol{p}'\cdot\boldsymbol{q}'}}{(2\pi\hbar)^{d}} V_i(\boldsymbol{q}')\end{aligned} \tag{S.III.20}$$

and by performing the Fourier transform on both sides:

$$\begin{aligned}\psi_{j'}(\boldsymbol{q}) = {}& \int_{\mathbb{R}^d} \mathrm{d}^d p' \frac{e^{\frac{i}{\hbar}\boldsymbol{p}'\cdot\boldsymbol{q}}}{(2\pi\hbar)^{d/2}} \tilde{\psi}_{j'}(\boldsymbol{p}') = \int_{\mathbb{R}^d} \mathrm{d}^d p' \frac{e^{\frac{i}{\hbar}\boldsymbol{p}'\cdot\boldsymbol{q}}}{(2\pi\hbar)^{d/2}} \delta^d(\boldsymbol{p}-\boldsymbol{p}')\,\delta_{jj'} + \\ &+ \int_{\mathbb{R}^d} \mathrm{d}^d p' \frac{e^{\frac{i}{\hbar}\boldsymbol{p}'\cdot\boldsymbol{q}}}{(2\pi\hbar)^{d/2}} \frac{1}{E - E_{\boldsymbol{p}'} - \mathcal{E}_{j'} + i\varepsilon} \sum_k \sum_i \left(\langle j' \mid \chi_i\rangle \langle \chi_i \mid k\rangle\right) \int_{\mathbb{R}^d} \mathrm{d}^d q' \psi_k(\boldsymbol{q}') \frac{e^{-\frac{i}{\hbar}\boldsymbol{p}'\cdot\boldsymbol{q}'}}{(2\pi\hbar)^{d/2}} V_i(\boldsymbol{q}') = \\ &= \frac{e^{\frac{i}{\hbar}\boldsymbol{p}\cdot\boldsymbol{q}}}{(2\pi\hbar)^{d/2}} \delta_{jj'} + \\ &+ \sum_k \sum_i \left(\langle j' \mid \chi_i\rangle \langle \chi_i \mid k\rangle\right) \int_{\mathbb{R}^d} \mathrm{d}^d p' \frac{1}{E - E_{\boldsymbol{p}'} - \mathcal{E}_{j'} + i\varepsilon} \int_{\mathbb{R}^d} \mathrm{d}^d q' \psi_k(\boldsymbol{q}') \frac{e^{-\frac{i}{\hbar}\boldsymbol{p}'\cdot(\boldsymbol{q}'-\boldsymbol{q})}}{(2\pi\hbar)^{d}} V_i(\boldsymbol{q}')\end{aligned} \tag{S.III.21}$$

Taking a few simplifications for the model:

1. **Delta interaction**: Introduced as $H_{int} = \sum_{i\in\{1,2,3\}} V_i \delta(\mathbf{q}-\mathbf{q_i})|\chi_i\rangle\langle\chi_i|$. Choosing $\mathbf{q} = \mathbf{q}_{i'}$, i.e. in the position of the sites, gives:

$$\begin{aligned}\psi_{j'}\left(\boldsymbol{q}_{i'}\right) =& \frac{e^{\frac{i}{\hbar}\boldsymbol{p}\cdot\boldsymbol{q}_{i'}}}{(2\pi\hbar)^{d/2}}\delta_{jj'}+\\ &+\sum_k\sum_i\left(\mathcal{V}_i\left\langle j' \mid \chi_i\right\rangle\left\langle\chi_i \mid k\right\rangle\right)\psi_k\left(\boldsymbol{q}_i\right)\int\limits_{\mathbb{R}^d}\mathrm{d}^d p'\frac{1}{E_{\boldsymbol{p}}+\mathcal{E}_j-E_{\boldsymbol{p}'}-\mathcal{E}_{j'}+i\varepsilon}\frac{e^{-\frac{i}{\hbar}\boldsymbol{p}'\cdot\left(\boldsymbol{q}_i-\boldsymbol{q}_{i'}\right)}}{(2\pi\hbar)^d}\end{aligned}\tag{S.III.22}$$

where we used that $E = E_{\mathbf{p}} + \mathcal{E}_j$. The equation for the wavefunction has now become a set of N^2 linear equations, that for each j and given some $\mathbf{p}$, we solve for the vector $\vec{\psi}\left(\boldsymbol{q}_{i'}\right)$. Having the solutions at hand, we plug them into the T matrix elements equation:

$$\begin{aligned}\left\langle\boldsymbol{p}'j'\left|T\right|\boldsymbol{p}j\right\rangle &= \sum_k\sum_i\left(\left\langle j' \mid \chi_i\right\rangle\left\langle\chi_i \mid k\right\rangle\right)\int\limits_{\mathbb{R}^d}\mathrm{d}^d q'\psi_k\left(\boldsymbol{q}'\right)\frac{e^{-\frac{i}{\hbar}\boldsymbol{p}'\cdot\boldsymbol{q}'}}{(2\pi\hbar)^{d/2}}\mathcal{V}_i\delta\left(\boldsymbol{q}'-\boldsymbol{q}_i\right) =\\ &= \sum_k\sum_i\left(\mathcal{V}_i\left\langle j' \mid \chi_i\right\rangle\left\langle\chi_i \mid k\right\rangle\right)\psi_k\left(\boldsymbol{q}_i\right)\frac{e^{-\frac{i}{\hbar}\boldsymbol{p}'\cdot\boldsymbol{q}_i}}{(2\pi\hbar)^{d/2}}\end{aligned}\tag{S.III.23}$$

2. **Short separation**: Choosing $\mathbf{q}_i = 0$ gives:

$$\begin{aligned}\psi_{j'} &= \frac{1}{(2\pi\hbar)^{d/2}}\delta_{jj'}+\frac{1}{(2\pi\hbar)^d}\sum_k v_{j'k}\psi_k\int\limits_{\mathbb{R}^d}\mathrm{d}^d p'\frac{1}{E_{\boldsymbol{p}}+\mathcal{E}_j-E_{\boldsymbol{p}'}-\mathcal{E}_{j'}+i\varepsilon} =\\ &= \frac{1}{(2\pi\hbar)^{d/2}}\delta_{jj'}+\frac{1}{(2\pi\hbar)^d}\left(v\vec{\psi}\right)_{j'}\int\limits_{\mathbb{R}^d}\mathrm{d}^d p'\frac{1}{E_{\boldsymbol{p}}+\mathcal{E}_j-E_{\boldsymbol{p}'}-\mathcal{E}_{j'}+i\varepsilon}\end{aligned}\tag{S.III.24}$$

where we write $\psi_{j'} = \psi_{j'}(0)$ and define the interaction matrix v and as in the main text $v_{kl} = \sum_{i=1}^{3}\langle k|\chi_i\rangle V_i\langle\chi_i|l\rangle$, $k,l \in \{+,0,-\}$. This reduces the number of equations by a factor of N - the number of sites.

3. **One dimension**: Taking $d = 1$ we can evaluate the integral using Cauchy's integral formula (without the renormalization required for higher dimensions), closing a contour in the upper half of the complex plane, and then taking $\varepsilon \to 0^+$:

$$\int\limits_{-\infty}^{\infty} dp'\frac{1}{E-E_{p'}-\mathcal{E}_{j'}+i\varepsilon} = \int\limits_{-\infty}^{\infty} dp'\frac{2m}{2m\left(E-\mathcal{E}_{j'}\right)-p^2+i\varepsilon} = -\frac{i\pi\sqrt{2m}}{\sqrt{E-\mathcal{E}_{j'}}}\tag{S.III.25}$$

Finally, the T-matrix elements take the form:

$$\left\langle p'j'\left|T\left(E\right)\right|pj\right\rangle = \frac{1}{\sqrt{2\pi\hbar}}\sum_k v_{j'k}\psi_k\left(E\right)\tag{S.III.26}$$

where ψ is given by:

$$\psi_{j'}\left(E\right) = \frac{1}{\sqrt{2\pi\hbar}}\delta_{jj'}-\frac{1}{2\pi\hbar}\frac{i\pi\sqrt{2m}}{\sqrt{E-\mathcal{E}_{j'}}}\sum_k v_{j'k}\psi_k\left(E\right)\tag{S.III.27}$$

thus having:

$$\left\langle p'j'\left|T\left(E\right)\right|pj\right\rangle = i\frac{\sqrt{E-\mathcal{E}_{j'}}}{\pi\sqrt{2m}}\left(\sqrt{2\pi\hbar}\psi_{j'}\left(E\right)-\delta_{jj'}\right)\tag{S.III.28}$$

The interaction matrix v for the toy model is given by:

$$v_{k\ell} = \sum_i\mathcal{V}_i\left\langle k \mid \chi_i\right\rangle\left\langle\chi_i \mid \ell\right\rangle = \begin{cases} w & k=\ell\\ u & (k,\ell) = (-,0),(0,+),(+,-)\\ u^* & (k,\ell) = (-,+),(+,0),(0,-)\end{cases}\tag{S.III.29}$$

with

$$w = \frac{1}{3}\left(\mathcal{V}_1 + \mathcal{V}_2 + \mathcal{V}_3\right)$$
$$u = \frac{1}{3}\left(\mathcal{V}_1 + \mathcal{V}_2 e^{i\frac{2\pi}{3}} + \mathcal{V}_3 e^{-i\frac{2\pi}{3}}\right) \tag{S.III.30}$$

The equation for the wave function can be written in matrix form. This is allowed since the vector $\psi_{j'}$ is computed separately for any j. Therefore, we can define the matrix Ψ with both indices that satisfies the following:

$$\Psi_{j'j}(E) = \frac{1}{\sqrt{2\pi\hbar}}\delta_{j'j} - \frac{i}{2\hbar}\frac{\sqrt{2m}}{\sqrt{E-\mathcal{E}_{j'}}}\sum_k v_{j'k}\Psi_{kj}(E) = \frac{\delta_{j'j}}{\sqrt{2\pi\hbar}} + \left(D_1(E)\, v\Psi(E)\right)_{j'j} \tag{S.III.31}$$

$$\Rightarrow \Psi(E) = \frac{1}{\sqrt{2\pi\hbar}}\left(1 - D_1(E)\, v\right)^{-1} \tag{S.III.32}$$

where we define $\left(D_1(E)\right)_{j'j} = -\frac{i}{2\hbar}\frac{\sqrt{2m}}{\sqrt{E-\mathcal{E}_{j'}}}\delta_{j'j}$.
The T matrix is given by:

$$T(E) = D_2(E)\left(\left(1 - D_1(E)\, v\right)^{-1} - 1\right) \tag{S.III.33}$$

where $T_{j'j}(E) = \langle p'j' \left| T(E) \right| pj\rangle$ and $\left(D_2(E)\right)_{j'j} = \frac{i}{\pi}\frac{\sqrt{E-\mathcal{E}_{j'}}}{\sqrt{2m}}\delta_{j'j}$.

D. Explicit T matrix elements

The explicit form is $T_{ij}(E) = \frac{\tilde{T}_{ij}(E)}{D_T(E)}$ where:

$$\begin{aligned} D_T(E) = {} & i\pi m\sqrt{2m}\left(2\left(3\left(\Im(u)\right)^2 \Re(u) - \left(\Re(u)\right)^3\right) + 3\left|u\right|^2 w - w^3\right) + \\ & + 2\pi m\hbar\left(\left|u\right|^2 - w^2\right)\left(\sqrt{E-\mathcal{E}_-} + \sqrt{E-\mathcal{E}_0} + \sqrt{E-\mathcal{E}_+}\right) + \\ & + 2i\pi\hbar^2\sqrt{2m}w\left(\sqrt{E-\mathcal{E}_-}\sqrt{E-\mathcal{E}_0} + \sqrt{E-\mathcal{E}_-}\sqrt{E-\mathcal{E}_+} + \sqrt{E-\mathcal{E}_0}\sqrt{E-\mathcal{E}_+}\right) + \\ & + 4\pi\hbar^3\sqrt{E-\mathcal{E}_-}\sqrt{E-\mathcal{E}_0}\sqrt{E-\mathcal{E}_+} \end{aligned} \tag{S.III.34}$$

and:

$$\tilde{T}_{ij}(E) = \begin{cases} \sqrt{2}\hbar\sqrt{E-\mathcal{E}_i}\sqrt{E-\mathcal{E}_j}\left(u^*\sqrt{2}\hbar\sqrt{E-\mathcal{E}_{k\neq i,j}} + i\sqrt{m}\left(u^* w - u^2\right)\right) & q=0 \\ \sqrt{2}\hbar\sqrt{E-\mathcal{E}_i}\sqrt{E-\mathcal{E}_j}\left(u\sqrt{2}\hbar\sqrt{E-\mathcal{E}_{k\neq i,j}} + i\sqrt{m}\left(uw - (u^*)^2\right)\right) & q=1 \end{cases} \tag{S.III.35}$$

with $q=0$ for clockwise rates and $q=1$ for counterclockwise rates. Since we are interested in $\left|T_{ij}(E)\right|^2$, we look at $\left|\tilde{T}_{ij}(E)\right|^2$, but we should consider the value of which E takes, since $E < \mathcal{E}_+$ implies $\sqrt{E-\mathcal{E}_+} \in i\mathbb{R}$:
<u>$E > \mathcal{E}_+$</u>:

$$\begin{aligned} \left|\tilde{T}_{ij}(E)\right|^2 = {} & 2\hbar^2\left(E-\mathcal{E}_i\right)\left(E-\mathcal{E}_j\right)\Big(2\left|u\right|^2\hbar^2\left(E-\mathcal{E}_{k\neq i,j}\right) + m\left|u^* w - u^2\right|^2 \\ & + (-1)^{q_{ij}}\, 2\hbar\sqrt{2m}\sqrt{E-\mathcal{E}_{k\neq i,j}}\,\Im\left(u^3\right)\Big) \end{aligned} \tag{S.III.36}$$

<u>$\mathcal{E}_0 < E < \mathcal{E}_+$</u>: (we will see later this is relevant only for rates a_{0-}, a_{-0})

$$\begin{aligned} \left|\tilde{T}_{-0}(E)\right|^2 = \left|\tilde{T}_{0-}(E)\right|^2 = {} & 2\hbar^2\left(E-\mathcal{E}_-\right)\left(E-\mathcal{E}_0\right)\Big(2\left|u\right|^2\hbar^2\left(\mathcal{E}_+ - E\right) + m\left|u^* w - u^2\right|^2 \\ & + 2\hbar\sqrt{2m}\sqrt{\mathcal{E}_+ - E}\left(2\left|u\right|^2 w - \Re\left(u^3\right)\right)\Big) \end{aligned} \tag{S.III.37}$$

E. Violation of micro-reversibility

Micro-reversibility is governed by the difference (non zero for $E > \mathcal{E}_+$):

$$\begin{aligned} &\left|\langle \boldsymbol{p}', i\left|T\left(E\right)\right|\boldsymbol{p}, j\rangle\right|^2 - \left|\langle -\boldsymbol{p}, j\left|T\left(E\right)\right| - \boldsymbol{p}', i\rangle\right|^2 = \\ &= \left|\langle \boldsymbol{p}', i\left|T\left(E\right)\right|\boldsymbol{p}, j\rangle\right|^2 - \left|\langle \boldsymbol{p}, j\left|T\left(E\right)\right|\boldsymbol{p}', i\rangle\right|^2 = \\ &= \left(-1\right)^q \frac{\left(2\hbar\right)^3 \sqrt{2m}\left(E-\mathcal{E}_i\right)\left(E-\mathcal{E}_j\right)\sqrt{E-\mathcal{E}_{k\neq i,j}}}{\left|D_T\left(E\right)\right|^2} \Im\left(u^3\right) \end{aligned} \tag{S.III.38}$$

where $6\sqrt{3}\Im\left(u^3\right) = \left(\mathcal{V}_1 - \mathcal{V}_2\right)\left(\mathcal{V}_1 - \mathcal{V}_3\right)\left(\mathcal{V}_2 - \mathcal{V}_3\right)$.

IV. DECOMPOSITION OF THE RATES OF TRANSITION

Since the T matrix elements are computed for the on-shell energy of the ingoing state $E = E_p + \mathcal{E}_\ell$ (with $E_p = \frac{p^2}{2m}$), we can change the integration variable from p to E:

$$\begin{aligned} a_{k\ell} =& 2\nu\pi \int dp dp' \frac{e^{-\beta E_p}}{Z_P} \delta\left(E_{p'} + \mathcal{E}_k - E_p - \mathcal{E}_\ell\right)\left|\langle k, p'\left|T\right|\ell, p\rangle\right|^2 \\ =& \frac{2\pi\nu}{Z_P} \int_{\mathcal{E}_\ell}^{\infty} dE \int_{-\infty}^{\infty} dp'\, e^{-\beta\left(E-\mathcal{E}_\ell\right)} \frac{m}{\sqrt{2m\left(E-\mathcal{E}_\ell\right)}} \delta\left(E_{p'} + \mathcal{E}_k - E\right)\left|\langle p', k\left|T\right|p, \ell\rangle\right|^2 = \\ =& \frac{2\pi\nu}{Z_P} \int_{\mathcal{E}_\ell}^{\infty} dE \int_{-\infty}^{\infty} dp' e^{-\beta\left(E-\mathcal{E}_\ell\right)} \frac{m}{\sqrt{2m\left(E-\mathcal{E}_\ell\right)}} 2m\delta\left(p'^2 - 2m\left(E-\mathcal{E}_k\right)\right)\left|\langle p', k\left|T\right|p, \ell\rangle\right|^2 = \\ =& \frac{2\pi\nu}{Z_P} \int_{\mathcal{E}_\ell}^{\infty} dE \int_{-\infty}^{\infty} dp' e^{-\beta\left(E-\mathcal{E}_\ell\right)} \frac{2m^2}{\sqrt{2m\left(E-\mathcal{E}_\ell\right)}} \sum_{\alpha=\pm1} \left(\frac{\delta\left(p' + \alpha\sqrt{2m\left(E-\mathcal{E}_k\right)}\right)}{\sqrt{2m\left(E-\mathcal{E}_k\right)}}\right)\left|\langle p', k\left|T\right|p, \ell\rangle\right|^2 \end{aligned} \tag{S.IV.1}$$

The term inside the δ function implies $E > \mathcal{E}_k$, thus integration over p' gives:

$$a_{k\ell} = \frac{4\pi m\nu}{Z_P} \int_{\max\{\mathcal{E}_k, \mathcal{E}_\ell\}}^{\infty} dE e^{-\beta\left(E-\mathcal{E}_\ell\right)} \frac{\left|T_{k\ell}\left(E\right)\right|^2}{\sqrt{E-\mathcal{E}_k}\sqrt{E-\mathcal{E}_\ell}} \tag{S.IV.2}$$

We recall that inside the integration, p, p' are defined by E and ℓ, k, respectively. Hence we can write the T matrix as $T_{k\ell}\left(E\right) \equiv \langle p', k\left|T\left(E\right)\right|p, \ell\rangle$. Note that the partition function for a single free particle has: $Z_P^{-1} \propto \sqrt{\beta}$.

Denoting $\tilde{Z}_P = Z_P\sqrt{\beta}$, we have that $\tilde{Z}_P$ is independent of temprature. Thus, we define:

$$\begin{aligned} a_0 =& \frac{4\pi m\nu}{\tilde{Z}_P} \frac{2\hbar^2\sqrt{\left(E-\mathcal{E}_+\right)\left(E-\mathcal{E}_0\right)\left(E-\mathcal{E}_-\right)}}{\left|D_T\left(E\right)\right|^2} \left(2\left|u\right|^2\hbar^2\sqrt{E} + m\left|u^* w - u^2\right|^2 \frac{1}{\sqrt{E}}\right) \\ a_1 =& \frac{4\pi m\nu}{\tilde{Z}_P} \frac{2\hbar^2\sqrt{\left(E-\mathcal{E}_+\right)\left(E-\mathcal{E}_0\right)\left(E-\mathcal{E}_-\right)}}{\left|D_T\left(E\right)\right|^2} \left(2\sqrt{2m}\hbar\Im\left(u^3\right)\right) \\ b_{k\ell} =& \frac{4\pi m\nu}{\tilde{Z}_P} \frac{2\hbar^2\sqrt{\left(E-\mathcal{E}_+\right)\left(E-\mathcal{E}_0\right)\left(E-\mathcal{E}_-\right)}}{\left|D_T\left(E\right)\right|^2} \times \\ & \left(2\left|u\right|^2\hbar^2\left(\sqrt{E-\mathcal{E}_{n\neq k,\ell}} - \sqrt{E}\right) + m\left|u^* w - u^2\right|^2\left(\frac{1}{\sqrt{E-\mathcal{E}_{n\neq k,\ell}}} - \frac{1}{\sqrt{E}}\right)\right) \\ \tilde{b}_{k\ell} =& \begin{cases} \frac{4\pi m\nu}{\tilde{Z}_P} \frac{1}{\sqrt{\left(E-\mathcal{E}_-\right)\left(E-\mathcal{E}_0\right)}} \frac{\left|\tilde{T}_{12}(E)\right|^2}{\left|D_T(E)\right|^2} & k, \ell \in \{0, -\} \\ 0 & else \end{cases} \end{aligned} \tag{S.IV.3}$$

with these definitions, one arrives at the main text's expression in equation (6). Note that at high energies:

$$\frac{\sqrt{(E-\mathcal{E}_+)(E-\mathcal{E}_0)(E-\mathcal{E}_-)}}{|D_T(E)|^2} \approx \frac{1}{(4\pi\hbar^3)^2 E^{\frac{3}{2}}} \tag{S.IV.4}$$

So, in leading order:

$$\begin{aligned} a_0 &\propto \frac{|u|^2}{E} \\ a_1 &\propto \frac{\Im(u^3)}{E^{\frac{3}{2}}} \\ b_{k\ell} &\propto \frac{|u|^2 \mathcal{E}_{n\neq k,\ell}}{E^2} \end{aligned} \tag{S.IV.5}$$

Implying equation (7) in the main paper.

Additionally, with this decomposition we can write:

$$\tilde{a}_{k\ell} = \sqrt{\beta}\left(\int_{\mathcal{E}_+}^{\infty} dEe^{-\beta E}(a_0 + b_{k\ell}) + \int_{\mathcal{E}_0}^{\mathcal{E}_+} dEe^{-\beta E}\tilde{b}_{k\ell}\right) \tag{S.IV.6}$$

such that $a_{k\ell} = \tilde{a}_{k\ell} + (-1)^q \tilde{a}_1$ where $\tilde{a}_1 = \sqrt{\beta}\int_{\mathcal{E}_+}^{\infty} dEe^{-\beta E}a_1 \propto \Delta_{VMR}$, and $\tilde{a}_{k\ell} = \tilde{a}_{\ell k} > 0$ by definition. Hence, we can write:

$$\begin{aligned} c &= a_{-+}a_{+0}a_{0-} - a_{-0}a_{0+}a_{+-} = \\ &= e^{\beta\sum_\ell \mathcal{E}_\ell}\left((\tilde{a}_{-+}+\tilde{a}_1)(\tilde{a}_{+0}+\tilde{a}_1)(\tilde{a}_{0-}+\tilde{a}_1) - (\tilde{a}_{-0}-\tilde{a}_1)(\tilde{a}_{0+}-\tilde{a}_1)(\tilde{a}_{+-}-\tilde{a}_1)\right) = \\ &= e^{\beta\sum_\ell \mathcal{E}_\ell}\tilde{a}_1\left(\tilde{a}_1^2 + \tilde{a}_{-+}\tilde{a}_{+0} + \tilde{a}_{-+}\tilde{a}_{0-} + \tilde{a}_{+0}\tilde{a}_{0-}\right) \\ &= e^{\beta\sum_\ell \mathcal{E}_\ell}\frac{\tilde{a}_1}{\Delta_{VMR}}\Delta_{VMR}\left((\Delta_{VMR})^2\left(\frac{\tilde{a}_1}{\Delta_{VMR}}\right)^2 + \tilde{a}_{-+}\tilde{a}_{+0} + \tilde{a}_{-+}\tilde{a}_{0-} + \tilde{a}_{+0}\tilde{a}_{0-}\right) \\ &= e^{\beta\sum_\ell \mathcal{E}_\ell}\left(\frac{\tilde{a}_1}{\Delta_{VMR}}\right)^3\Delta_{VMR}\left((\Delta_{VMR})^2 + \underbrace{\left(\frac{\Delta_{VMR}}{\tilde{a}_1}\right)^2 \tilde{a}_{-+}\tilde{a}_{+0} + \tilde{a}_{-+}\tilde{a}_{0-} + \tilde{a}_{+0}\tilde{a}_{0-}}_{c_0}\right) \end{aligned} \tag{S.IV.7}$$

Since $\tilde{a}_1 \propto \Delta_{VMR}$, this expression is well defined for any finite non-zero temperature, with c_0 not directly dependent on Δ_{VMR}.

V. SUFFICIENT CONDITION FOR OSCILLATIONS AT THE HIGH-TEMPERATURE LIMIT

The condition for oscillations is $\gamma < 0$. Using equation (6) from the main text, while denoting $A_i = \int_{\mathcal{E}_+}^{\infty} dEe^{-\beta E}a_i$, $B_{n\neq\{k,\ell\}} = \int_{\mathcal{E}_+}^{\infty} dEe^{-\beta E}b_{k\ell}$, and $\tilde{B}_{n\neq\{k,\ell\}} = \int_{\mathcal{E}_+}^{\infty} dEe^{-\beta E}\tilde{b}_{k\ell}$, then the condition $\gamma < 0$ becomes:

$$\begin{aligned} 0 > &\left(2A_0\sum_i e^{\beta\mathcal{E}_i} + B_-\left(e^{\beta\mathcal{E}_0} + e^{\beta\mathcal{E}_+}\right) + B_0\left(e^{\beta\mathcal{E}_-} + e^{\beta\mathcal{E}_+}\right) + \left(B_+ + \tilde{B}_+\right)\left(e^{\beta\mathcal{E}_-} + e^{\beta\mathcal{E}_0}\right)\right)^2 - \\ &-4\left(\prod_i e^{\beta\mathcal{E}_i}\right)\left(\sum_j e^{-\beta\mathcal{E}_j}\right)\left(3A_0^2 + 2A_0\left(B_- + B_0 + B_+ + \tilde{B}_+\right) + A_1^2 + B_-B_0 + (B_- + B_0)\left(B_+ + \tilde{B}_+\right)\right) \end{aligned} \tag{S.V.1}$$

In the limit of high temperatures $\beta \to 0$ A_0 diverges as $\Gamma(0, \beta E_{A_0})$, with some $E_{A_0} > \mathcal{E}_+$, and Γ being the incomplete gamma function $\Gamma(s,t) = \int_t^{\infty} dxe^{-x}x^{s-1}$. This divergence is regulated in the expression of the rates since: $\lim_{\beta\to 0}\sqrt{\beta}\Gamma(0,\beta E_{A_0}) = 0$. Similarly, when taking the limit in (S.V.1), A_0 is multiplied by a term linearly converging to 0. Therefore, when taking $\beta \to 0$ the condition for oscillations becomes:

$$-3A_1^2 + (B_- - B_0)^2 + (B_- - B_+)(B_0 - B_+) + \tilde{B}_+\left(\tilde{B}_+ + (B_+ - B_0) + (B_+ - B_-)\right) < 0 \tag{S.V.2}$$

In order to arrive at the expression given in (8) in the main text, we look into the expansion of the $b_{k\ell}$ terms. We have for $\mathcal{E}_i < \mathcal{E}_j$:

$$B_i - B_j = \frac{4\pi m\nu}{\tilde{Z}_P} \int_{\mathcal{E}_+}^{\infty} \frac{2\hbar^2 \sqrt{(E-\mathcal{E}_+)(E-\mathcal{E}_0)(E-\mathcal{E}_-)}}{|D_T|^2} \left(\frac{2|u|^2 \hbar^2}{2\sqrt{E}} (\mathcal{E}_j - \mathcal{E}_i) + \mathcal{O}\left(E^{-\frac{3}{2}}\right) \right) \tag{S.V.3}$$

Since for $E > \mathcal{E}_+$ we have: $\frac{2|u|^2\hbar^2}{2\sqrt{E}} (\mathcal{E}_j - \mathcal{E}_i) \leq \frac{2|u|^2\hbar^2}{2\sqrt{\mathcal{E}_+}} (\mathcal{E}_j - \mathcal{E}_i)$, then we define:

$$\begin{aligned} \Delta B'_{ij} &= B_i - B_j + \frac{4\pi m\nu}{\tilde{Z}_P} \int_{\mathcal{E}_+}^{\infty} \frac{2\hbar^2 \sqrt{(E-\mathcal{E}_+)(E-\mathcal{E}_0)(E-\mathcal{E}_-)}}{|D_T|^2} \left(\frac{2|u|^2 \hbar^2}{2\sqrt{\mathcal{E}_+}} (\mathcal{E}_j - \mathcal{E}_i) - \frac{2|u|^2 \hbar^2}{2\sqrt{E}} (\mathcal{E}_j - \mathcal{E}_i) \right) = \\ &= B_i - B_j + \frac{|u|^2 \hbar}{\Im(u^3)} \frac{\mathcal{E}_j - \mathcal{E}_i}{2\sqrt{2m\mathcal{E}_+}} A_1 - \frac{4\pi m\nu}{\tilde{Z}_P} \int_{\mathcal{E}_+}^{\infty} \frac{2\hbar^2 \sqrt{(E-\mathcal{E}_+)(E-\mathcal{E}_0)(E-\mathcal{E}_-)}}{|D_T|^2} \left(\frac{2|u|^2 \hbar^2}{2\sqrt{E}} (\mathcal{E}_j - \mathcal{E}_i) \right) \end{aligned} \tag{S.V.4}$$

s.t. $\Delta B'_{ij} \geq B_i - B_j$. With this we can write:

$$\begin{aligned} &-3A_1^2 + (B_- - B_0)^2 + (B_- - B_+)(B_0 - B_+) + \tilde{B}_+ \left(\tilde{B}_+ + (B_+ - B_0) + (B_+ - B_-) \right) \leq \\ &\leq -3A_1^2 + \left(\Delta B'_{-0}\right)^2 + \Delta B'_{-+} \Delta B'_{0+} + \tilde{B}_+ \left(\tilde{B}_+ + (B_+ - B_0) + (B_+ - B_-) \right) = \\ &= -3A_1^2 + A_1^2 \left(\frac{|u|^2 \hbar}{\Im(u^3)} \frac{1}{2\sqrt{2m\mathcal{E}_+}} \right)^2 \left((\mathcal{E}_- - \mathcal{E}_0)^2 + (\mathcal{E}_- - \mathcal{E}_+)(\mathcal{E}_0 - \mathcal{E}_+) + \mathcal{E}_+ \mathcal{E}_{low-energy} \right) = \\ &= -3A_1^2 + 3\frac{A_1^2}{\mathcal{E}_{VDB}} \left(\frac{(\mathcal{E}_- - \mathcal{E}_0)^2 + (\mathcal{E}_- - \mathcal{E}_+)(\mathcal{E}_0 - \mathcal{E}_+)}{\mathcal{E}_+} + \mathcal{E}_{low-energy} \right) \end{aligned} \tag{S.V.5}$$

where we define:

$$\begin{aligned} &\mathcal{E}_{low-energy} = \\ &= \frac{\mathcal{E}_{VDB}}{3A_1^2} \left(\left(\Delta B'_{-0}\right)^2 + \Delta B'_{-+} \Delta B'_{0+} + \tilde{B}_+ \left(\tilde{B}_+ + (B_+ - B_0) + (B_+ - B_-) \right) \right) - \frac{(\mathcal{E}_- - \mathcal{E}_0)^2 + (\mathcal{E}_- - \mathcal{E}_+)(\mathcal{E}_0 - \mathcal{E}_+)}{\mathcal{E}_+} \end{aligned} \tag{S.V.6}$$

Notice that if the r.h.s of Eq. (S.V.5) is negative, then the oscillation condition, Eq. (S.V.2), automatically holds. Rearranging (S.V.5) we get equation (8) in the main text. Additionally, $\mathcal{E}_{VDB}, A_1^2 \propto \Delta_{VMR}$, thus making $\mathcal{E}_{VDB}/A_1^2$ generally non-zero at points where DB is kept. In order to understand the quantity $\mathcal{E}_{low-energy}$ better, we plot $\mathcal{E}_{low-energy}/\frac{(\mathcal{E}_- - \mathcal{E}_0)^2 + (\mathcal{E}_- - \mathcal{E}_+)(\mathcal{E}_0 - \mathcal{E}_+)}{\mathcal{E}_+}$ (see Figure 1). When this ratio is small, equation (8) simplifies to:

$$\mathcal{E}_{VDB} > \frac{(\mathcal{E}_- - \mathcal{E}_0)^2 + (\mathcal{E}_- - \mathcal{E}_+)(\mathcal{E}_0 - \mathcal{E}_+)}{\mathcal{E}_+}. \tag{S.V.7}$$

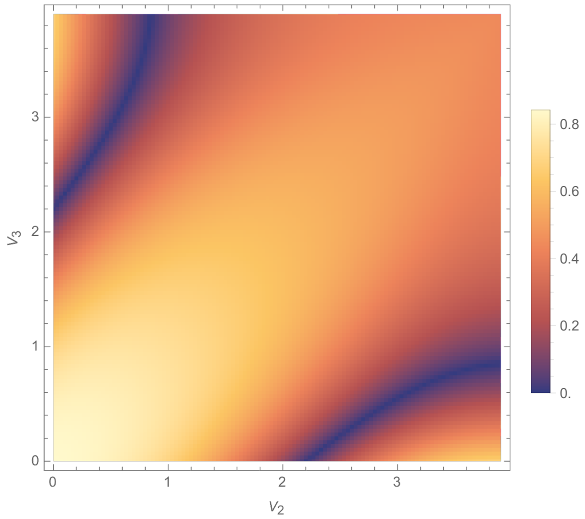

FIG. 1. $E_{low-energy}/\frac{(\mathcal{E}_{-}-\mathcal{E}_0)^2+(\mathcal{E}_{-}-\mathcal{E}_{+})(\mathcal{E}_0-\mathcal{E}_{+})}{\mathcal{E}_{+}}$ as a function of V_2 (x-axis) and V_3 (y-axis). Parameters: $\tau = 1.85$, $\phi = 0.575$, $V_1 = 6$. $\hbar = k_B = m = 1$. Max $\Delta\epsilon = \mathcal{E}_{+} - \mathcal{E}_{-}$.

VI. PROOF FOR NO OSCILLATIONS IN THE LOW-TEMPERATURE LIMIT

For low temperatures, two main results explain the behavior of the rates in the limit of $\beta \to \infty$:

1.
$$\lim_{\beta\to\infty} \frac{a_{k\ell}}{a_{\ell k}} = \begin{cases} \infty & \mathcal{E}_\ell > \mathcal{E}_k \\ 0 & \mathcal{E}_\ell < \mathcal{E}_k \end{cases} \tag{S.VI.1}$$

This means that the transition rates from a higher energetic state to a lower energetic state are much greater than the rates of the reverse process.

2. For $\mathcal{E}_i < \mathcal{E}_j, \mathcal{E}_k < \mathcal{E}_\ell$:
$$\frac{B^L(E_0)}{B^U} \le \lim_{\beta\to\infty} \frac{a_{k\ell}}{a_{ij}} \le \frac{B^U}{B^L(E_0)} \tag{S.VI.2}$$

with $0 < B^L(E_0), B^U < \infty$. This means that rates of transition from a higher energy level to a lower one are not significantly bigger or smaller than one another.
Combining both results we find that for $\mathcal{E}_i < \mathcal{E}_j, \mathcal{E}_\ell < \mathcal{E}_k$:
$$\lim_{\beta\to\infty} \frac{a_{k\ell}}{a_{ij}} = 0 \tag{S.VI.3}$$

i.e., all transition rates from a low energetic state to a higher one are significantly smaller than any rate from a high energetic state to a lower one.

Using this result we have: $\mathcal{E}_k < \mathcal{E}_\ell$:

$$\begin{aligned} a^{LT}_{k\ell} &\equiv \lim_{\beta\to\infty} \frac{a_{k\ell}}{\omega_{dis}} = \lim_{\beta\to\infty} \frac{a_{k\ell}}{\sum_{i\neq j} a_{ij}} = \lim_{\beta\to\infty} \left(\sum_{\mathcal{E}_i<\mathcal{E}_j} \frac{a_{ij}}{a_{k\ell}} + \sum_{\mathcal{E}_i>\mathcal{E}_j} \frac{a_{ij}}{a_{k\ell}} \right)^{-1} = \\ &= \left(\sum_{\mathcal{E}_i<\mathcal{E}_j} \lim_{\beta\to\infty} \frac{a_{ij}}{a_{k\ell}} \right)^{-1} > 0 \end{aligned} \tag{S.VI.4}$$

and similarly $\lim_{\beta\to\infty} \frac{a_{\ell k}}{\omega_{dis}} = 0$. A full proof that $\mathrm{sign}(\gamma(0)) = 1$ at the limit of $\beta \to \infty$ will follow the proofs of (S.VI.1) and (S.VI.2).

Before proving (S.VI.1) and (S.VI.2), we'll see that the rates of transition converge to zero in the limit of $\beta \to \infty$ for this model. The rates of transition $a_{k\ell}$ are dependent of temperature by factor

$\sqrt{\beta}e^{-\beta(E-\mathcal{E}_\ell)}$ where we integrate over $E > \mathcal{E}_\ell$. The integral converges for any finite non-zero value of β, since $\sqrt{\beta}e^{-\beta(E-\mathcal{E}_\ell)}$ is monotonically decreasing for $\beta > \frac{1}{2(E-\mathcal{E}_\ell)}$, then by dominant convergence theorem we can exchange integration and the limit $\beta \to \infty$. At the limit:

$$\lim_{\beta\to\infty} \sqrt{\beta}e^{-\beta(E-\mathcal{E}_\ell)} = 0 \tag{S.VI.5}$$

for any $E > \mathcal{E}_\ell$, hence $\lim_{\beta\to\infty} a_{k\ell} = 0$

For the proof of (S.VI.1) and (S.VI.2) we define:

$$f_{k\ell}(E) = \frac{1}{\sqrt{E - \max\{\mathcal{E}_\ell, \mathcal{E}_k\}}} \frac{|T_{k\ell}(E)|^2}{\sqrt{(E-\mathcal{E}_\ell)(E-\mathcal{E}_k)}} \tag{S.VI.6}$$

By using the explicit T matrix expressions, we find that for $E \geq \max\{\mathcal{E}_\ell, \mathcal{E}_k\}$, this function is bounded, and that $f_{k\ell} > 0$.
Thus, we'll denote some arbitrary $E_0 > \mathcal{E}_+$, and define upper and lower bounds for the function:

$$\begin{aligned} B^L_{k\ell}(E_0) &= \min_{E\in[\max\{\mathcal{E}_\ell,\mathcal{E}_k\},E_0]} f_{k\ell}(E) \\ B^U_{k\ell} &= \max_{E\geq\max\{\mathcal{E}_\ell,\mathcal{E}_k\}} f_{k\ell}(E) \end{aligned} \tag{S.VI.7}$$

From that we'll define common bounds:

$$\begin{aligned} B^L(E_0) &= \min_{k\neq\ell} B^L_{k\ell}(E_0) \\ B^U &= \max_{k\neq\ell} B^U_{k\ell} \end{aligned} \tag{S.VI.8}$$

Thus we have $0 < B^L(E_0) \leq B^U < \infty$. Defining:

$$\bar{a}_{k\ell} = \int\limits_{\max\{\mathcal{E}_\ell,\mathcal{E}_k\}}^{\infty} dE e^{-\beta E}\sqrt{E - \max\{\mathcal{E}_\ell, \mathcal{E}_k\}} f_{k\ell}(E) \tag{S.VI.9}$$

gives:

$$B^L(E_0) \int\limits_{\max\{\mathcal{E}_\ell,\mathcal{E}_k\}}^{E_0} dE e^{-\beta E}\sqrt{E - \max\{\mathcal{E}_\ell, \mathcal{E}_k\}} \leq \bar{a}_{k\ell} \leq B^U \int\limits_{\max\{\mathcal{E}_\ell,\mathcal{E}_k\}}^{\infty} dE e^{-\beta E}\sqrt{E - \max\{\mathcal{E}_\ell, \mathcal{E}_k\}} \tag{S.VI.10}$$

changing integration variable $x = E - \max\{\mathcal{E}_\ell, \mathcal{E}_k\}$

$$B^L(E_0) e^{-\beta\max\{\mathcal{E}_\ell,\mathcal{E}_k\}} \int\limits_0^{E_0-\max\{\mathcal{E}_\ell,\mathcal{E}_k\}} dE e^{-\beta E}\sqrt{x} \leq \bar{a}_{k\ell} \leq B^U e^{-\beta\max\{\mathcal{E}_\ell,\mathcal{E}_k\}} \int\limits_0^{\infty} dx e^{-\beta E}\sqrt{x} \tag{S.VI.11}$$

Now using:

$$\int\limits_0^A dE e^{-\beta E}\sqrt{x} = \frac{\sqrt{\pi}\mathrm{erf}\left(\sqrt{\beta A}\right) - 2\sqrt{\beta A}e^{-A\beta}}{2\sqrt{\beta^3}} \tag{S.VI.12}$$

with erf being the error function, we get

$$B^L(E_0) e^{-\beta\max\{\mathcal{E}_\ell,\mathcal{E}_k\}} \frac{\sqrt{\pi}\mathrm{erf}\left(\sqrt{\beta E_0^{k\ell}}\right) - 2\sqrt{\beta E_0^{k\ell}}e^{-E_0^{k\ell}\beta}}{2\sqrt{\beta^3}} \leq \bar{a}_{k\ell} \leq B^U e^{-\beta\max\{\mathcal{E}_\ell,\mathcal{E}_k\}} \frac{\sqrt{\pi}}{2\sqrt{\beta^3}} \tag{S.VI.13}$$

where $E_0^{k\ell} = E_0 - \max\{\mathcal{E}_\ell, \mathcal{E}_k\} > 0$.
Since $\frac{a_{k\ell}}{a_{ij}} = e^{\beta(\mathcal{E}_\ell-\mathcal{E}_j)}\frac{\bar{a}_{k\ell}}{\bar{a}_{ij}}$:

$$e^{\beta(\mathcal{E}_\ell-\mathcal{E}_j)}\frac{e^{-\beta\max\{\mathcal{E}_\ell,\mathcal{E}_k\}}}{e^{-\beta\max\{\mathcal{E}_i,\mathcal{E}_j\}}}\xi_{k\ell}(\beta) \leq \frac{a_{k\ell}}{a_{ij}} \leq e^{\beta(\mathcal{E}_\ell-\mathcal{E}_j)}\frac{e^{-\beta\max\{\mathcal{E}_\ell,\mathcal{E}_k\}}}{e^{-\beta\max\{\mathcal{E}_i,\mathcal{E}_j\}}}\left(\xi_{ij}(\beta)\right)^{-1} \tag{S.VI.14}$$

where

$$\xi_{k\ell}(\beta) \equiv \frac{B^L(E_0)}{B^U} \frac{\sqrt{\pi}\text{erf}\left(\sqrt{\beta E_0^{k\ell}}\right) - 2\sqrt{\beta E_0^{k\ell}} e^{-E_0^{k\ell}\beta}}{\sqrt{\pi}} \tag{S.VI.15}$$

By the properties of the error function, at the limit of low temperatures:

$$\lim_{\beta\to\infty} \xi_{k\ell}(\beta) = \frac{B^L(E_0)}{B^U} \tag{S.VI.16}$$

To finish up the proof, we consider the two cases presented before:

1. $i = \ell, j = k$, i.e., considering rates between two states in both directions:

$$e^{\beta(\mathcal{E}_\ell - \mathcal{E}_k)} \xi_{k\ell}(\beta) \le \frac{a_{k\ell}}{a_{\ell k}} \le e^{\beta(\mathcal{E}_\ell - \mathcal{E}_k)} (\xi_{k\ell}(\beta))^{-1} \tag{S.VI.17}$$

   and at the limit of $\beta \to \infty$ we arrive at (S.VI.1), with exponential convergence (divergence)

2. $i < j, k < \ell$, i.e., considering rates of transition from a higher energy level to a lower one:

$$\xi_{k\ell}(\beta) \le \frac{a_{k\ell}}{a_{ij}} \le (\xi_{ij}(\beta))^{-1} \tag{S.VI.18}$$

   which in the limit becomes (S.VI.2)

Finally, we look into the transition rate matrix and note that if we define: $\tilde{M} = \frac{1}{\omega_{dis}} M$ then if $\tilde{M}$ has strictly real eigenvalues then M has only real eigenvalues. Since we have at the limit:

$$\lim_{\beta\to\infty} \tilde{M}_{ij} = \begin{cases} a_{ij}^{LT} & i < j \\ 0 & i > j \end{cases} \tag{S.VI.19}$$

then $\tilde{M}$ is triangular. Its eigenvalues are the diagonal elements which are real $\Rightarrow$ M eigenvalues are real $\Rightarrow$ no oscillations in low temperatures.

VII. N - LEVEL SYSTEM TOY MODEL

A larger number of oscillations during the thermalization time implies a more significant difference between the oscillatory and non-oscillatory decay, potentially simplifying the possibility of experimentally distinguishing between these two dynamics. Mathematically, the oscillation number is defined as the maximal value of $\left|\frac{\Im[\lambda_i]}{\Re[\lambda_i]}\right|$, where λ_i is an eigenvalue of M. The maximum number of oscillations we show in the main text for the three-level toy model is ~ 0.16. As shown below, this number can be increased using the following procedure. We consider an N-level system and concentrate on oscillations that involve all its energy levels, that is, $|1\rangle \to |2\rangle \to ... \to |N\rangle \to |1\rangle$. The oscillation strength will be maximized if the oscillation in the opposite direction and oscillations among other levels are forbidden, i.e., $a_{1,2} = a_{2,3} = ... = a_{N-1,N} = a_{N,1} = 0$. Moreover, for $a_{21} = a_{3,2} = ... = a_{N,N-1} = a_{1,N} = a$ the oscillation number is maximized. Notice that this situation corresponds to the strongest violation of detailed balance where a_{ik}/a_{ki} is either zero or infinite.

In this case, the M matrix is

$$M = a \begin{pmatrix} -1 & 1 & 0 & ... & 0 \\ 0 & -1 & 1 & \ddots & 0 \\ \vdots & \ddots & \ddots & \ddots & \vdots \\ 0 & \ddots & \ddots & -1 & 1 \\ 1 & 0 & \dots & 0 & -1 \end{pmatrix} \tag{S.VII.1}$$

This is a circulant matrix. The eigenvalues of this matrix are well-known [2] . In particular, the maximal rate between the imaginary and real part of its eigenvalues is $Cot[\frac{\pi}{N}]$. For large N, it can be approximated as N/π (see figure **??**). This shows that the oscillation number can be increased to any desired value by increasing the size of the system and maximizing the violation of detailed balance (see Eq. (S.VII.1)).

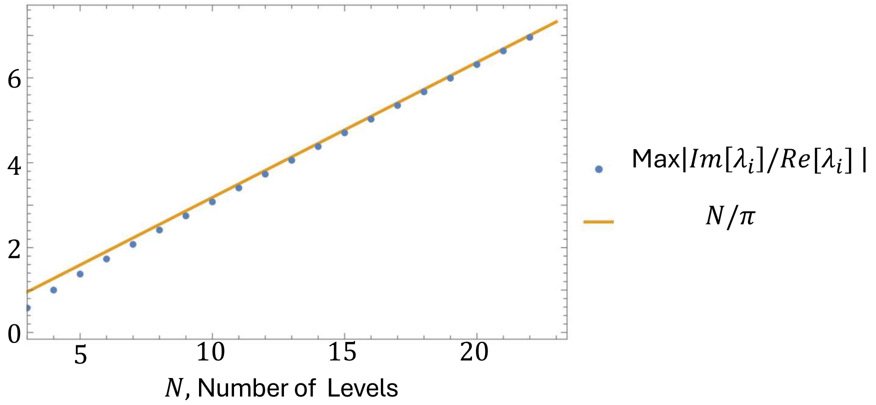

FIG. 2. Maximal oscillation number as a function of the level number for maximum violation of detailed balance. Eq. (S.VII.1) shows the form of the transition matrix.

REFERENCES

[1] R Dümcke. "The low density limit for an N-level system interacting with a free bose or fermi gas". In: *Communications in Mathematical Physics* 97.3 (Sept. 1985), pp. 331–359. DOI: 10.1007/BF01213401.

[2] Robert M Gray et al. "Toeplitz and circulant matrices: A review". In: *Foundations and Trends® in Communications and Information Theory* 2.3 (2006), pp. 155–239.

[3] John Russell Taylor. *Scattering theory: The quantum theory of nonrelativistic collisions*. New York: Wiley, 1972.
