# Supplementary material for: Thermalization without Detailed Balance: Population Oscillations in the Absence of Coherences
Source: J Phys Chem Lett. 2025 Apr 16;16(16):4066–71. doi: 10.1021/acs.jpclett.5c00499 (PMC12035852; doi:10.1021/acs.jpclett.5c00499)
Supplement: Supplementary file 2 — jz5c00499_si_002.pdf [file jz5c00499_si_002.pdf]

jz-2025-00499c.R1

Name: Peer Review Information for "Thermalization without detailed balance: population oscillations in the absence of coherences"

First Round of Reviewer Comments

Reviewer: 1

Comments to the Author

Manuscript discusses evolution of open quantum system in the absence of without detailed balance (e.g. in presence of magnetic field). Authors employ Pauli rate equations (Redfield/Lindblad quantum master equation) to evolve the system. Analysis of the Liouvillian indicates presence of exceptional points which separate monotonic and oscillatory behavior in population dynamics when evolving towards thermal equilibrium. Overall, within its approximations the consideration is solid. So, the manuscript deserves to be published.

There are several points of potential confusion authors may want to consider before the publication:

1. In the abstract authors claim: “Open quantum systems that complywith detailed balance decay in a non-oscillatory manner to thermal equilibrium.” This statement is misleading, because it is true only within approximations resulting in the Redfield/Lindblad QME. The approximation is quite drastic, so that resulting evolution is not accurate. In general, even with presence of detiled balance system evolution may demonstrate oscillatory behavior [see, e.g., J. Chem. Phys. **160**, 044116 (2024)].
2. On p.4 authors make a distinction between systems with and withoutexternal driving present. The latter distinction is not always well defined. For example, when harmonic external driving is treated within the RWA, transformation into rotating frame of the field

ylieds timeindependent Hamiltonian. That is, system with and without external driving describe the same model in different frames. Such are, e.g., Bloch equations.

3. Another distinction authors make is between systems with and without coherences present when consideration is made within the system eigenbasis. This statement is also misleading in a sense that it is well defined within the realm of the Redfield/Lindblad QME level of distinction, which in many cases is not accurate even at weak system-bath coupling [see, e.g., arXiv:2412.12624]. In reality, bath always introduces coherences in the system. Moreover, the coherences will be time-non-local.

4. It would be helpful to notify the reader that the consideration andanalysis (including the very concept of exceptional points) is an approximation, which is quite limited in its applicability to description of open systems (strictly speaking, the method is stochastic classical approach).

Reviewer: 2

Comments to the Author

The manuscript investigates an open quantum system that violates detailed balance and is strongly coupled to a thermal environment, but includes no bath-induced coherences. The authors show that the thermalization dynamics of the system can exhibit oscillations in the populations of the eigenstates of the decoupled system. Intriguingly, these oscillations appear at high temperatures, and the regimes characterizing them are separated by exceptional points in the Liouvillian.

The work is novel and should be of interest to physical chemists working in fields ranging from the development of optical sensors to quantum transport. I am therefore pleased to recommend publication. Some optional comments and questions for the author are included below.

1. The alternating use of VDB as both a noun and a verb, while having a certain charm, is grammatically bold. Perhaps the authors should reconsider it? One possibility is saying e.g. “VDBing systems”.

2. There is a seeming contradiction here, because the author notes that “For systems that are weakly coupled… a_kl obeys DB” (citing Alicki's book; I believe it should refer only to systems coupled to a single bath at equilibrium). Yet, the treatment in the paper is all master equations, thereby only generally being valid at weak coupling. I suggest that the authors either modify this statement or justify the validity of master equations in the present context, especially because there is only a single thermal bath here (probably this is in reference 38?).

3. Figure 1 is in 2D, but all the results are in 1D. This is a little confusing, and the authors should consider changing the figure or at the very least noting this in the caption.

4. It would be useful to write down the complete Hamiltonian of the system before the GKLS approximation is applied, including the quantum dots, the free particle gas and the interaction.

5. The theory for the free particles is all apparently done at the single particle level, but it looks from equation (4) like they are assumed to be distinguishable. Does it perhaps not matter at the low density limit whether they obey Fermi or Bose statistics? I did not find an explicit statement about it in the manuscript.

6. It almost sounds like one should be able to extract work from a single bath here, because we see transient circular currents with a well-defined directionality. Could the authors comment on this?

7. In this context, could the authors comment on the relationship with "phaseonium"? See https://doi.org/10.1126/science.1078955.

Author's Response to Peer Review Comments:

We thank both referees for their comments. Below, we present the referee's report with our answers to each of their comments.

Referee 1

Manuscript discusses evolution of open quantum system in the absence of without detailed balance (e.g. in presence of magnetic field). Authors employ Pauli rate equations (Redfield/Lindblad quantum master equation) to evolve the system. Analysis of the Liouvillian indicates presence of exceptional points which separate monotonic and oscillatory behavior in population dynamics when evolving towards thermal equilibrium. Overall, within its approximations the consideration is solid. So, the manuscript deserves to be published.

> We thank the referee for the appreciation of our manuscript and the recommendation for publication. The referee's comments have helped to make the

manuscript more precise and avoid potential confusion. Below, we answer each of the referee's points

There are several points of potential confusion authors may want to consider before the publication:

1. In the abstract authors claim: "Open quantum systems that comply with detailed balance decay in a non-oscillatory manner to thermal equilibrium." This statement is misleading, because it is true only within approximations resulting in the Redfield/Lindblad QME. The approximation is quite drastic, so that resulting evolution is not accurate. In general, even with presence of detiled balance system evolution may demonstrate oscillatory behavior [see, e.g., J. Chem. Phys. 160, 044116 (2024)].

   We thank the referee for bringing this point to our attention. We have now clarified this point in the abstract and in the introduction. They now read:

   "Open quantum systems that comply with the master equation and detailed balance decay in a non-oscillatory manner to thermal equilibrium…"

   "In this letter, we use the Lindblad equation to study the thermalization of a nondegenerate N-level open quantum system that violates DB"

2. On p.4 authors make a distinction between systems with and without external driving present. The latter distinction is not always well defined. For example, when harmonic external driving is treated within the RWA, transformation into rotating frame of the field ylieds timeindependent Hamiltonian. That is, system with and without external driving describe the same model in different frames. Such are, e.g., Bloch equations.

   We point out that our work focuses on non-driven systems. We mention the case of driven systems because this is one of the types of systems that people have studied in the literature. In those works, the presence of the driving has been used to break detailed balance even in the presence of a single bath. The breaking of detailed balance plays a key role in the presence of LEP in the Pauli equation. It was only recently found that detailed balance with a single bath can also be broken without driving and we use this result to find exceptional points in this type of setup.

   We agree with the referee that by changing frames one could "hide" the Hamiltonian time dependence. But still, from a thermodynamic perspective, a non-driven and driven system behave very differently in the lab frame. For example, the stationary state of a non-driven, non-degenerate system interacting with a low-density gas will

be a thermal state. If the system is driven, the stationary state can diverge from the thermal state. We believe that this difference also affects the presence of exceptional points.

3. Another distinction authors make is between systems with and without coherences present when consideration is made within the system eigenbasis. This statement is also misleading in a sense that it is well defined within the realm of the Redfield/Lindblad QME level of distinction, which in many cases is not accurate even at weak system-bath coupling [see, e.g., arXiv:2412.12624]. In reality, bath always introduces coherences in the system. Moreover, the coherences will be time-nonlocal.

We agree with the referee that the lack of coherences is an approximation valid specifically in the realm of the Redifield/Lindblad QME. In order to clarify this point, we have now added the following clarification to the introduction:

"In this letter, we use the Lindblad equation to study the thermalization of a nondegenerate N-level open quantum system that violates DB"

As mentioned above, in the abstract, we have clarified that we are focusing on dynamics governed by the master equation.

4. It would be helpful to notify the reader that the consideration and analysis (including the very concept of exceptional points) is an approximation, which is quite limited in its applicability to description of open systems (strictly speaking, the method is stochastic classical approach).

We agree with the referee that the methods we use only approximately describe the dynamics. In order to clarify this, we have emphasized the approximate nature of the Lindblad equation:

> "The reduced dynamics follows the Gorini, Kossakowski, Lindblad and Sudarshan (GKLS) equation [30,31]. The latter is **approximately** valid in the weak coupling, low-density or singular coupling limit"

Referee 2

The manuscript investigates an open quantum system that violates detailed balance and is strongly coupled to a thermal environment, but includes no bathinduced coherences. The authors show that the thermalization dynamics of the system can exhibit oscillations in the populations of the eigenstates of the decoupled system. Intriguingly, these oscillations appear at high temperatures, and the regimes characterizing them are separated by exceptional points in the
Liouvillian.

The work is novel and should be of interest to physical chemists working in fields ranging from the development of optical sensors to quantum transport. I am therefore pleased to recommend publication. Some optional comments and questions for the author are included below.

We thank the referee for the appreciation of our work and all the comments and questions. Below, we answer all the questions and describe the changes that we introduced to the manuscript.

1. The alternating use of VDB as both a noun and a verb, while having a certain charm, is grammatically bold. Perhaps the authors should reconsider it? One possibility is saying e.g. “VDBing systems”.

   We thank the reviewer for bringing this point to our attention. We think part of the confusion is that in the previous manuscript we used VDB to represent two different terms: 1) violation of detailed balance at equilibrium; 2) violate detailed balance at equilibrium.

   We have now corrected this, and in the current version, VDB only refers to the violation of detailed balance at equilibrium.

2. There is a seeming contradiction here, because the author notes that “For systems that are weakly coupled… a_kl obeys DB” (citing Alicki's book; I believe it should refer only to systems coupled to a single bath at equilibrium). Yet, the treatment in the paper is all master equations, thereby only generally being valid at weak coupling. I suggest that the authors either modify this statement or justify the validity of master equations in the present context, especially because there is only a single thermal bath here (probably this is in reference 38?).

   Let us bring to the attention of the referee that the master equation can be derived in several limits, the weak coupling limit being only one of them. At the

weak coupling, in the presence of a single thermal bath and without driving, DB is automatically fulfilled. Another limit where the master equation is valid is the low-density limit. In this case, the interaction can go beyond the weak coupling and DB can be violated (see Alicki book and 38). The low density limit is the limit that is used in this work calculations.

3. Figure 1 is in 2D, but all the results are in 1D. This is a little confusing, and the authors should consider changing the figure or at the very least noting this in the caption.

We thank the referee for bringing this point to our attention. We have followed the referee's suggestion and added the following statement to the figure caption:

"In this work, we assume a short separation between the three quantum dots and a 1D gas. These assumptions allow us to calculate analytically the T-matrix elements."

4. It would be useful to write down the complete Hamiltonian of the system before the GKLS approximation is applied, including the quantum dots, the free particle gas and the interaction.

We thank the referee for this suggestion. But the results in the first pages of the manuscript are completely general as long as the evolution is given by a Pauli rate equation (1). As we explain above Eq 1, this equation holds for any non-degenerate system in the weak, low-density or singular coupling limit. Besides these conditions, our results are entirely general. We believe that including a particular Hamiltonian so early in the manuscript may mislead the reader into thinking that the results are only valid for that specific Hamiltonian, thereby misrepresenting the generality of our results.

5. The theory for the free particles is all apparently done at the single particle level, but it looks from equation (4) like they are assumed to be distinguishable. Does it perhaps not matter at the low density limit whether they obey Fermi or Bose statistics? I did not find an explicit statement about it in the manuscript.

We completely agree with the referee. In order to clarify this point, we have added the following clarification after the low-density limit is mentioned:

"At this limit, the gas statistic does not play any role"

6. It almost sounds like one should be able to extract work from a single bath here, because we see transient circular currents with a well-defined directionality. Could the authors comment on this?

   Although these oscillations may be used for transient work extraction, it is based on the resources present in the initial state. The lack of steady work extraction ensures compliance with the second law of thermodynamics. We have added the following point below equation 2 when the oscillatory behaviour is mentioned

   > "Although these oscillations may be used for a transient work extraction, it is based on the resources present in the initial state. The lack of steady work extraction ensures compliance with the second law of thermodynamics;"

7. In this context, could the authors comment on the relationship with "phaseonium"? See https://eur01.safelinks.protection.outlook.com/?url=https%3A%2F%2Fprotect.checkpoint.com%2Fv2%2Fr02%2F___https%3A%2F%2Fdoi.org%2F10.1126%2Fscience.1078955___.YzJlOnRlY2huaW9uOmM6bzphNTRmZDlhYWU5ZWJmZjk3YmQwNWRiMTQ4Y2M1NDcxYTo3OjhkZjE6MjYxZDMxODA2ZmM2NzgwZTZmY2FmYjFlNDQ3NGQ4ZjExNjQyMjFhMDY1NGU0ODA1NmVkN2FlM2U5OTVlOGY1ZTpwOlQ6Tg&data=05%7C02%7Cdgelbi%40technion.ac.il%7C58f7faeed7a44880e6db08dd6df1b1ba%7Cf1502c4cee2e411c9715c855f6753b84%7C1%7C0%7C638787607029373849%7CUnknown%7CTWFpbGZsb3d8eyJFbXB0eU1hcGkiOnRydWUsIlYiOiIwLjAuMDAwMCIsIlAiOiJXaW4zMiIsIkFOIjoiTWFpbCIsIldUIjoyfQ%3D%3D%7C0%7C%7C%7C&sdata=eFsLfQUL%2FtmIjhM%2FQibVn600gQPSHVgZgnVA8DvJIwA%3D&reserved=0.

   We believe that our results and the phaseonium work mentioned above represent two distinct effects. We focus on the thermalization of nondegenerate (also not nearly degenerate) systems. Our results are entirely independent of quantum coherences. In contrast, the phasenium work studies the work extraction of nearly degenerate systems. Moreover, for phasenium, quantum coherences play a crucial role. Finally, it is unclear whether one can obtain LEP with phasenium.
